# Crystal structure of a dinuclear ruthenium(II) complex with a bent CO_2_^2−^ bridge

**DOI:** 10.1107/S2056989018009921

**Published:** 2018-07-13

**Authors:** Tsugiko Takase, Ryosuke Abe, Dai Oyama

**Affiliations:** a Institute of Environmental Radioactivity, Fukushima University, 1 Kanayagawa, Fukushima 960-1296, Japan; b Graduate School of Science and Engineering, Fukushima University, 1 Kanayagawa, Fukushima 960-1296, Japan; c Department of Industrial Systems Engineering, Cluster of Science and Engineering, Fukushima University, 1 Kanayagawa, Fukushima 960-1296, Japan

**Keywords:** crystal structure, dinuclear ruth­en­ium(II) complex, carbon dioxide, carbonite ligand: bipyridyl ligand

## Abstract

The mol­ecular and crystal structures of a CO_2_^2−^-bridged dinuclear ruthenium complex is reported, namely, μ-carbonito-κ^2^ *C*:*O*-bis­[bis(2,2′-bi­pyridine-κ^2^ *N*,*N*′)carbon­ylruthenium(II)] bis­(hexa­fluorido­phosphate)–aceto­nitrile–diethyl ether (1/1/0.5), [Ru_2_(CO)_2_(C_10_H_8_N_2_)_4_(*μ:κ* ^2^-*C:O*-CO_2_)](PF_6_)_2_·CH_3_CN·0.5C_4_H_10_O. The complex cation in the title compound consists of two {Ru(CO)(bpy)_2_}^2+^ units (bpy = 2,2′-bi­pyridine) singly bridged by a μ:κ^2^-*C*:*O* carbonite anion, resulting in an unsymmetrical dinuclear structure. Some of the inter­atomic C⋯O distances involving the carbonyl ligands are shorter than the sum of the van der Waals radii. There are intra­molecular C—H⋯O and aromatic π–π contacts in the cationic complex. In the crystal, the cations are linked by pairs of C—H⋯F hydrogen bonds in addition to weak C—H⋯F inter­actions between the solvent mol­ecules and PF_6_^−^ counter-anions. The equatorial F atoms of one of the PF_6_^−^ anions are disordered over two sets of sites with an occupancy ratio of 0.908 (7):0.092 (7) while the central O atom of the diethyl ether solvent mol­ecule is disordered over an inversion centre.

## Abstract 2

In the complex cation of the title compound, two Ru^II^ atoms are bridged *via* the carbon and oxygen atoms of an anionic CO_2_^2–^ carbonite ligand, resulting in an unsymmetrical dinuclear structure.

## Chemical context   

Carbon dioxide is an undesirable by-product of the burning of fossil fuels and hence a significant pollutant responsible for climate change. There is considerable inter­est in using CO_2_ as a renewable energy source, capturing and reducing its atmospheric concentration to yield carbon-neutral fuels. However, because CO_2_ is thermodynamically stable, its activation and conversion to useful chemicals or fuels are challenging. At present, particular attention has been paid to transition metal catalysts for the activation of CO_2_ (Vogt *et al.*, 2018 ▸). An understanding of the mol­ecular and crystal structures and vibrational spectroscopic properties of CO_2_ ligands bonded to transition metal catalysts is essential because these reveal information concerning the inter­mediates of the catalytic activation of CO_2_ (Gibson, 1999 ▸).

Many transition metal compounds containing CO_2_ or derivatives thereof have been isolated and identified so far. CO_2_ ligands can coordinate not only in *κ* ^1^-*C* and *κ* ^2^-*C*,*O* modes in mononuclear complexes, but also in bridging modes (Gibson, 1996 ▸, 1999 ▸). A binuclear complex containing a bridging CO_2_ ligand is bonded to one metal by carbon and bonded to the other metal center by one (μ:κ^2^ mode) or two oxygen (μ:κ^3^ mode) atoms. Although bridging CO_2_ complexes can be synthesized in various ways, a particularly unusual method is the formation of anionic CO_2_^2−^-bridged dimers by the action of water and oxygen on a ruthenium complex containing an unstable formyl ligand (Gibson *et al.*, 1996 ▸). This formyl complex can be obtained from the corresponding dicarbonyl precursor (Toyohara *et al.*, 1995 ▸). Therefore, we used this convenient method to synthesize a dimer directly from the stable dicarbonyl precursor and further clarified the crystal structure of the solvated dimer.
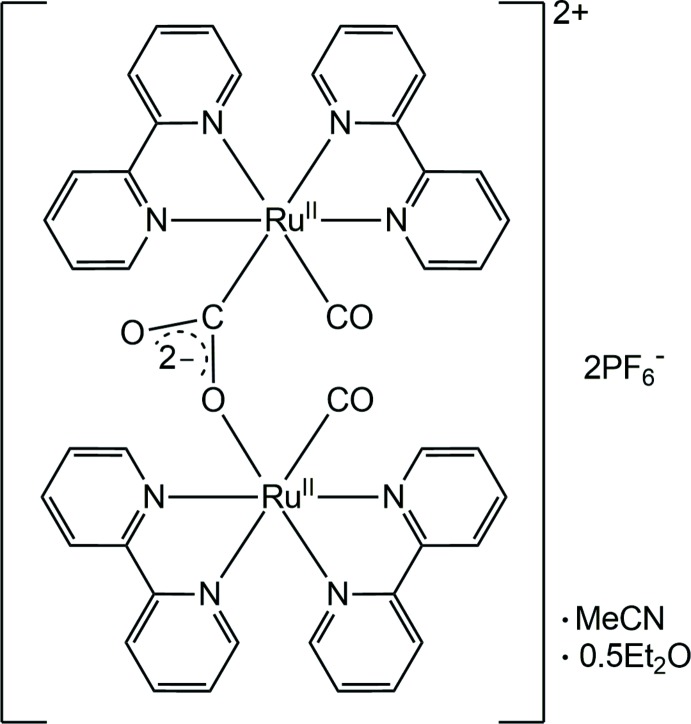


## Structural commentary   

**Figure 1:**
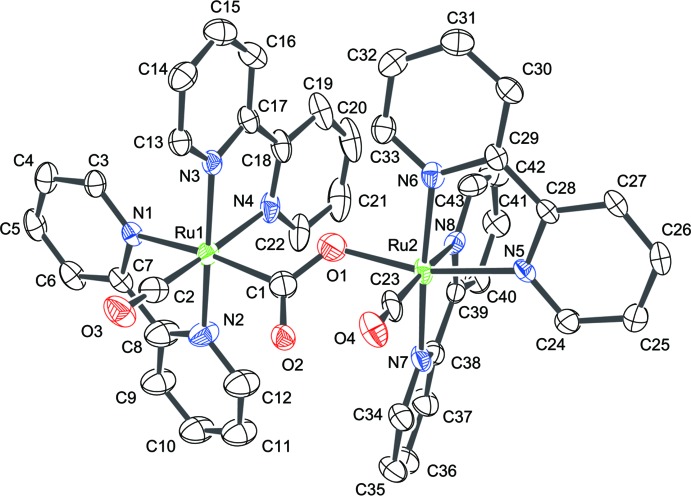
The mol­ecular structure of the complex cation in the title compound, with atom labels and displacement ellipsoids for non-H atoms drawn at the 50% probability level.

An X-ray structural analysis of the solvent-free dimer [Ru_2_(CO)_2_(C_10_H_8_N_2_)_4_(μ:κ^2^-*C*,*O*-CO_2_)]^2+^ has previously been performed by Gibson *et al.* (1996 ▸). In their model, the CO_2_^2−^-bridged anion was disordered in both the PF_6_^−^ and BPh_4_^−^ salts, which is not the case here. The title compound consists of two {Ru(CO)(bpy)_2_}^2+^ units (bpy = 2,2′-bi­pyridine) singly bridged by a μ:κ^2^-*C*,*O* carbonite ion, leading to an unsymmetrical dinuclear structure for the resulting cation (Fig. 1 ▸, Table 1 ▸). The coordination environment around each Ru^II^ atom is approximately octa­hedral, and the two terminal CO groups point in the same direction. The Ru1—N1 bond, which is *trans* to the carbonite carbon, is relatively long [2.154 (4) Å], suggesting a strong *trans* influence of the CO_2_^2−^ anion. Although the O—C—O angle in the anionic CO_2_^2−^ bridge [122.4 (5)°] has a typical value observed for this type of bridging anion (Gibson *et al.*, 1997 ▸, 1998 ▸), the lengths of the two C—O bonds [1.269 (9) Å for C1—O1 and 1.254 (7) Å for C1—O2] are almost identical with the difference (Δ = 0.015 Å) being much smaller than those of analogous singly anionic CO_2_-bridged Ru^II^ dimers (0.065 and 0.084 Å; Gibson *et al.*, 1997 ▸, 1998 ▸). The inter­atomic C2⋯O2 and C23⋯O2 distances between carbonyl ligands of 2.853 (6) and 2.818 (7) Å, respectively, are notably shorter than the sum of the van der Waals radii for the atoms involved. Additionally, there are intra­molecular C—H⋯O and aromatic π–π contacts, with a centroid-to-centroid distance of 3.889 (3) Å present in the complex cation (Table 2 ▸). These inter­actions may contribute to the unusual C—O bond-length distribution in the bridging CO_2_^2−^ anion described above.

The vibrational spectra of the terminal carbonyl groups are useful indicators of the electronic states around the central metal atoms or cations in metal complexes (Oyama *et al.*, 2009 ▸). The introduction of the anionic CO_2_^2−^ ligand into the {Ru(CO)(bpy)_2_}^2+^ unit results in a large redshift (*ca* 100 cm^−1^) for the C≡O group in the IR spectrum, which suggests significant differences in the electron density around the Ru^II^ cations. This IR band indicates that the carbonite ion has a strong electron-donating ability compared to those of the terminal carbonyl ligands.

## Supra­molecular features   

**Figure 2:**
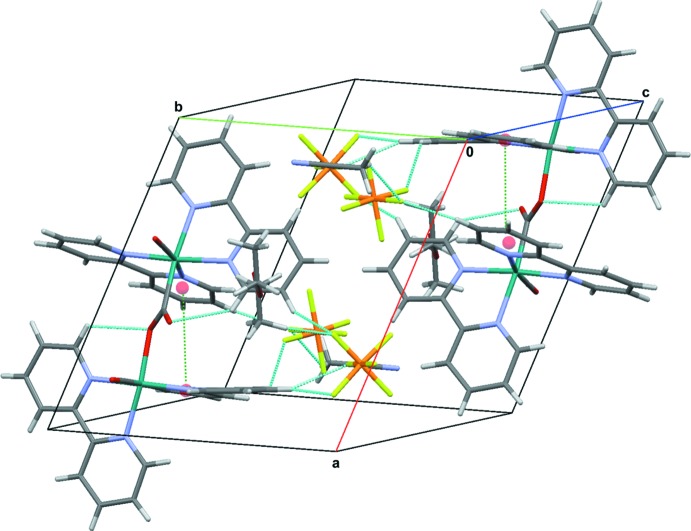
The crystal packing of the title compound. C—H⋯O and C—H⋯F hydrogen bonds (blue) and π–π contacts (green) are shown as dashed lines (for numerical details, see Table 2 ▸). Ring centroids are shown as red spheres. Only the major component of the disordered PF_6_^−^ anion is shown.

In the crystal structure, additional solvent mol­ecules are incorporated, *viz*. an acetro­nitrile and a disordered diethyl ether mol­ecule (occupancy 0.5) per formula unit. There are weak C—H⋯F and C—H⋯O hydrogen bonds between the complex cation and/or the solvent mol­ecules (CH_3_CN and Et_2_O) and the PF_6_^−^ anions, leading to the formation of a three-dimensional supra­molecular network structure (Table 2 ▸, Fig. 2 ▸).

## Database survey   

For related diruthenium complexes with a bent μ:κ^2^-*C*,*O* carbonite ion of the form [Ru_2_ *L*_2_ *L′*_2_(CO)_2_(μ:κ^2^-CO_2_)]^2+^, only one structure with the combination *L* = bpy and *L′* = 1,10-phenanthroline has been reported (Gibson *et al.*, 1998 ▸), although an analogue bearing both bpy and 2,2′:6′,2′′-terpyridine supporting ligands has also been described (Gibson *et al.*, 1997 ▸). Meanwhile, the structure of a diruthenium complex with a metallacyclic CO_2_-bridged anion has been determined by Arikawa *et al.* (2005 ▸).

## Synthesis and crystallization   

Although the solvent-free dimer had previously been prepared from the formyl complex (*cis*-[Ru(bpy)_2_(CO)(CHO)]^+^) and spectroscopically characterized (Gibson *et al.*, 1996 ▸), we used an alternative one-pot method starting from *cis*-[Ru(bpy)_2_(CO)_2_]^2+^ to prepare the title complex. The starting material, [Ru(bpy)_2_(CO)_2_](PF_6_)_2_, was prepared according to a literature method (Nagao *et al.*, 1994 ▸). [Ru(bpy)_2_(CO)_2_](PF_6_)_2_ (10 mg, 0.013 mmol) was dissolved in CH_3_CN (1 ml), followed by the addition of aqueous NaBH_4_ (2 eq.) at 253 K. The reaction mixture was stirred for 2 d, and then an excess of Et_2_O was added to the solution at the same temperature. Yellow–orange single crystals gradually formed from the solution when it was allowed to stand at 253 K, yielding X-ray quality crystals. The crystals were obtained in 48% yield (4 mg). The spectroscopic data for the solvent-free compound are consistent with those of Gibson *et al.* (1996 ▸).

## Refinement   

Crystal data, data collection and structure refinement details are summarized in Table 3 ▸. All hydrogen atoms were placed at calculated positions (C—H = 0.95–0.99 Å) and refined using a riding model with *U*_iso_(H) = 1.2*U*_eq_(C). The equatorial F atoms of one of the PF_6_^−^ anions are disordered over two sets of sites with an occupancy ratio of 0.908 (7):0.092 (7). The minor components were refined with isotropic displacement parameters. The same applies for the diethyl ether solvent mol­ecule, the central O atom of which is disordered over an inversion centre. The maximum and minimum residual electron density peaks of 3.14 and 2.41 e Å^−3^ are located 0.77 and 0.73 Å, respectively, from atom Ru1.

## Supplementary Material

Crystal structure: contains datablock(s) General, I. DOI: 10.1107/S2056989018009921/wm5452sup1.cif

Structure factors: contains datablock(s) I. DOI: 10.1107/S2056989018009921/wm5452Isup2.hkl

Supporting information file. DOI: 10.1107/S2056989018009921/wm5452Isup4.mol

CCDC reference: 1855027

Additional supporting information:  crystallographic information; 3D view; checkCIF report

## Figures and Tables

**Table 1:** Selected bond lengths (Å)

Ru1—N1	2.154 (4)	Ru2—O1	2.097 (4)
Ru1—N2	2.095 (5)	Ru2—N5	2.103 (4)
Ru1—N3	2.055 (5)	Ru2—N6	2.069 (5)
Ru1—N4	2.124 (5)	Ru2—N7	2.068 (5)
Ru1—C1	2.068 (6)	Ru2—N8	2.125 (4)
Ru1—C2	1.867 (6)	Ru2—C23	1.837 (6)

**Table 2:** Hydrogen-bond geometry (Å, °)

*D*—H⋯*A*	*D*—H	H⋯*A*	*D*⋯*A*	*D*—H⋯*A*
C5—H3⋯F5*A* ^i^	0.95	2.54	3.390 (8)	149
C5—H3⋯F5*B* ^i^	0.95	2.18	2.91 (4)	133
C6—H4⋯F4*B* ^ii^	0.95	2.52	2.88 (3)	102
C11—H7⋯F6*A*	0.95	2.44	3.269 (8)	145
C12—H8⋯O2	0.95	2.49	3.241 (9)	136
C13—H9⋯O2^iii^	0.95	2.39	3.105 (6)	132
C19—H13⋯F4*A* ^iv^	0.95	2.29	3.077 (9)	140
C21—H15⋯F7	0.95	2.30	3.237 (10)	170
C25—H18⋯F12^v^	0.95	2.44	3.130 (8)	129
C30—H21⋯O4^vi^	0.95	2.56	3.473 (7)	162
C33—H24⋯O1	0.95	2.47	3.035 (7)	118
C36—H27⋯F1	0.95	2.40	3.292 (8)	156
C36—H27⋯F3*A*	0.95	2.53	3.322 (9)	141
C36—H27⋯F3*B*	0.95	2.48	3.16 (4)	128
C37—H28⋯F11	0.95	2.49	3.205 (9)	132
C42—H31⋯F5*A* ^iv^	0.95	2.50	3.320 (8)	145
C43—H32⋯F2^iv^	0.95	2.48	3.223 (7)	135
C44—H34⋯F7	0.98	2.54	3.318 (10)	136
C47—H39⋯F6*A* ^v^	0.98	2.30	3.18 (3)	148
C47—H39⋯F3*B* ^v^	0.98	2.39	3.19 (4)	139

Symmetry codes: (i) 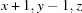; (ii) 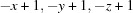; (iii) 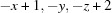; (iv) 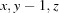; (v) 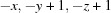; (vi) 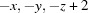.

**Table 3:** Experimental details

Crystal data	
Chemical formula	[Ru_2_(C_43_H_32_N_8_)](PF_6_)_2_·C_2_H_3_N·0.5C_4_H_10_O
*M*_r_	1294.96
Crystal system, space group	Triclinic, *P* 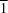
Temperature (K)	93
*a*, *b*, *c* (Å)	13.3151 (3), 13.9878 (3), 14.9621 (3)
α, β, γ (°)	77.3797 (7), 89.7109 (7), 65.3536 (7)
*V* (Å^3^)	2459.95 (8)
*Z*	2
Radiation type	Mo *K*α
μ (mm^−1^)	0.78
Crystal size (mm)	0.20 × 0.20 × 0.20

Data collection	
Diffractometer	Rigaku Saturn70
Absorption correction	Multi-scan (*REQAB*; Rigaku, 1998 ▸)
*T*_min_, *T*_max_	0.691, 0.855
No. of measured, independent and observed [*F* ^2^ > 2.0σ(*F* ^2^)] reflections	25892, 11139, 10548
*R*_int_	0.026
(sin θ/λ)_max_ (Å^−1^)	0.649

Refinement	
*R*[*F* ^2^ > 2σ(*F* ^2^)], *wR*(*F* ^2^), *S*	0.062, 0.140, 1.07
No. of reflections	11139
No. of parameters	674
No. of restraints	2
H-atom treatment	H-atom parameters constrained
Δρ_max_, Δρ_min_ (e Å^−3^)	3.14, −2.41

Computer programs: *PROCESS-AUTO* (Rigaku, 1998 ▸), *SIR97* (Altomare *et al.*, 1999 ▸), *SHELXL2017/1* (Sheldrick, 2015 ▸), *Mercury* (Macrae *et al.*, 2008 ▸), *ORTEP-3 for Windows* (Farrugia, 2012 ▸), *CrystalStructure* (Rigaku, 2010 ▸), *PLATON* (Spek, 2009 ▸) and *publCIF* (Westrip, 2010 ▸).

## supplementary crystallographic information

### Crystal data

**Table:**

[Ru_2_(CO_2_)(CO)_2_(C_10_H_8_N_2_)_4_](PF_6_)_2_·C_2_H_3_N·0.5C_4_H_10_O	*Z* = 2
*M**_r_* = 1294.96	*F*(000) = 1294.00
Triclinic, *P*1	*D*_x_ = 1.748 Mg m^−^^3^
Hall symbol: -P 1	Mo *K*α radiation, λ = 0.71075 Å
*a* = 13.3151 (3) Å	Cell parameters from 25072 reflections
*b* = 13.9878 (3) Å	θ = 3.0–27.5°
*c* = 14.9621 (3) Å	µ = 0.78 mm^−^^1^
α = 77.3797 (7)°	*T* = 93 K
β = 89.7109 (7)°	Prism, yellow-orange
γ = 65.3536 (7)°	0.20 × 0.20 × 0.20 mm
*V* = 2459.95 (8) Å^3^	

### Data collection

**Table:**

Rigaku Saturn70 diffractometer	10548 reflections with *F*^2^ > 2.0σ(*F*^2^)
Detector resolution: 7.143 pixels mm^-1^	*R*_int_ = 0.026
ω scans	θ_max_ = 27.5°
Absorption correction: multi-scan (*REQAB*; Rigaku, 1998)	*h* = −17→16
*T*_min_ = 0.691, *T*_max_ = 0.855	*k* = −18→18
25892 measured reflections	*l* = −19→19
11139 independent reflections	

### Refinement

**Table:**

Refinement on *F*^2^	Secondary atom site location: difference Fourier map
*R*[*F*^2^ > 2σ(*F*^2^)] = 0.062	Hydrogen site location: inferred from neighbouring sites
*wR*(*F*^2^) = 0.140	H-atom parameters constrained
*S* = 1.07	*w* = 1/[σ^2^(*F*_o_^2^) + (0.0352*P*)^2^ + 16.6385*P*] where *P* = (*F*_o_^2^ + 2*F*_c_^2^)/3
11139 reflections	(Δ/σ)_max_ = 0.010
674 parameters	Δρ_max_ = 3.14 e Å^−^^3^
2 restraints	Δρ_min_ = −2.41 e Å^−^^3^
Primary atom site location: structure-invariant direct methods	

### Special details

**Table:**

Refinement. Refinement was performed using all reflections. The weighted R-factor (wR) and goodness of fit (S) are based on F^2^. R-factor (gt) are based on F. The threshold expression of F^2^ > 2.0 sigma(F^2^) is used only for calculating R-factor (gt).

### Fractional atomic coordinates and isotropic or equivalent isotropic displacement parameters (Å^2^)

**Table:**

	*x*	*y*	*z*	*U*_iso_*/*U*_eq_	Occ. (<1)
Ru1	0.52423 (3)	0.06985 (3)	0.78277 (3)	0.02534 (10)	
Ru2	0.13834 (3)	0.15875 (3)	0.83500 (2)	0.02277 (10)	
P2	0.26348 (14)	0.42263 (13)	0.35883 (13)	0.0480 (4)	
F7	0.2612 (5)	0.3171 (4)	0.3370 (4)	0.0867 (17)	
F8	0.2670 (6)	0.5276 (5)	0.3765 (4)	0.0960 (18)	
F9	0.1372 (5)	0.4864 (5)	0.3299 (7)	0.147 (4)	
F10	0.3919 (4)	0.3542 (5)	0.3860 (4)	0.0977 (18)	
F11	0.2467 (6)	0.3838 (5)	0.4637 (4)	0.102 (2)	
F12	0.2855 (5)	0.4559 (4)	0.2547 (4)	0.0858 (16)	
O1	0.3046 (3)	0.0982 (3)	0.8060 (3)	0.0373 (8)	
O2	0.3448 (3)	0.1955 (3)	0.8859 (3)	0.0289 (7)	
O3	0.6125 (4)	0.0723 (4)	0.9660 (3)	0.0421 (9)	
O4	0.1746 (3)	0.1627 (3)	1.0313 (3)	0.0360 (8)	
O5	−0.532 (3)	0.527 (3)	0.004 (3)	0.170 (8)*	0.5000
N1	0.6809 (3)	0.0268 (3)	0.7245 (3)	0.0220 (7)	
N2	0.5206 (4)	0.2200 (4)	0.7176 (4)	0.0370 (10)	
N3	0.5353 (3)	−0.0846 (3)	0.8240 (3)	0.0239 (8)	
N4	0.4540 (4)	0.0538 (4)	0.6628 (3)	0.0329 (9)	
N5	−0.0295 (3)	0.1895 (3)	0.8445 (3)	0.0211 (7)	
N6	0.1481 (3)	0.0035 (3)	0.8716 (3)	0.0237 (8)	
N7	0.1208 (3)	0.3143 (3)	0.7788 (3)	0.0252 (8)	
N8	0.1115 (3)	0.1722 (3)	0.6920 (3)	0.0232 (8)	
N9	0.1126 (6)	0.5536 (6)	0.0057 (5)	0.0671 (17)	
C1	0.3716 (5)	0.1310 (4)	0.8342 (4)	0.0337 (11)	
C2	0.5793 (4)	0.0728 (4)	0.8962 (4)	0.0323 (11)	
C3	0.7617 (4)	−0.0736 (4)	0.7368 (3)	0.0271 (9)	
C4	0.8640 (4)	−0.0946 (5)	0.7046 (4)	0.0340 (11)	
C5	0.8851 (4)	−0.0088 (5)	0.6590 (4)	0.0353 (12)	
C6	0.8019 (5)	0.0960 (5)	0.6446 (4)	0.0328 (11)	
C7	0.7001 (4)	0.1120 (4)	0.6787 (3)	0.0259 (9)	
C8	0.6078 (6)	0.2196 (5)	0.6711 (5)	0.0472 (7)	
C9	0.6110 (6)	0.3155 (5)	0.6240 (5)	0.0472 (7)	
C10	0.5230 (6)	0.4126 (5)	0.6222 (5)	0.0472 (7)	
C11	0.4344 (6)	0.4134 (5)	0.6690 (5)	0.0472 (7)	
C12	0.4359 (6)	0.3156 (5)	0.7168 (5)	0.0472 (7)	
C13	0.5758 (4)	−0.1503 (4)	0.9081 (3)	0.0265 (9)	
C14	0.5847 (5)	−0.2545 (4)	0.9319 (4)	0.0339 (11)	
C15	0.5512 (5)	−0.2943 (5)	0.8665 (4)	0.0375 (12)	
C16	0.5085 (5)	−0.2273 (5)	0.7803 (4)	0.0341 (11)	
C17	0.4999 (4)	−0.1223 (4)	0.7600 (3)	0.0268 (9)	
C18	0.4525 (4)	−0.0444 (5)	0.6711 (4)	0.0325 (11)	
C19	0.4062 (5)	−0.0677 (6)	0.6007 (4)	0.0422 (13)	
C20	0.3576 (5)	0.0126 (7)	0.5206 (4)	0.0536 (18)	
C21	0.3583 (5)	0.1118 (7)	0.5129 (5)	0.0553 (18)	
C22	0.4078 (5)	0.1301 (5)	0.5851 (4)	0.0433 (14)	
C23	0.1626 (4)	0.1605 (4)	0.9552 (4)	0.0294 (10)	
C24	−0.1152 (4)	0.2870 (4)	0.8294 (4)	0.0298 (10)	
C25	−0.2233 (4)	0.3006 (4)	0.8376 (4)	0.0332 (11)	
C26	−0.2439 (4)	0.2101 (5)	0.8623 (4)	0.0306 (10)	
C27	−0.1563 (4)	0.1082 (4)	0.8763 (3)	0.0246 (9)	
C28	−0.0495 (4)	0.1000 (4)	0.8667 (3)	0.0205 (8)	
C29	0.0497 (4)	−0.0042 (4)	0.8803 (3)	0.0217 (8)	
C30	0.0472 (4)	−0.1047 (4)	0.8992 (3)	0.0273 (9)	
C31	0.1447 (5)	−0.1977 (4)	0.9102 (4)	0.0336 (11)	
C32	0.2439 (5)	−0.1887 (4)	0.9016 (4)	0.0340 (11)	
C33	0.2425 (4)	−0.0875 (4)	0.8816 (3)	0.0282 (10)	
C34	0.1292 (4)	0.3824 (4)	0.8264 (4)	0.0322 (10)	
C35	0.1314 (5)	0.4799 (5)	0.7838 (4)	0.0377 (12)	
C36	0.1253 (5)	0.5088 (5)	0.6891 (5)	0.0405 (13)	
C37	0.1152 (5)	0.4407 (4)	0.6389 (4)	0.0346 (11)	
C38	0.1114 (4)	0.3447 (4)	0.6854 (4)	0.0259 (9)	
C39	0.0990 (4)	0.2687 (4)	0.6373 (3)	0.0241 (9)	
C40	0.0742 (4)	0.2917 (4)	0.5431 (4)	0.0313 (10)	
C41	0.0641 (5)	0.2154 (5)	0.5042 (4)	0.0353 (11)	
C42	0.0803 (5)	0.1165 (5)	0.5596 (4)	0.0355 (11)	
C43	0.1041 (5)	0.0979 (4)	0.6526 (4)	0.0312 (10)	
C44	0.1336 (6)	0.3826 (6)	0.1280 (5)	0.0533 (16)	
C45	0.1221 (6)	0.4792 (6)	0.0593 (5)	0.0469 (14)	
C46	−0.479 (2)	0.5381 (18)	0.0804 (15)	0.202 (8)*	
C47	−0.369 (2)	0.4976 (19)	0.1086 (17)	0.235 (10)*	
H1	0.7479	−0.1327	0.7691	0.0325*	
H2	0.9191	−0.1669	0.7135	0.0408*	
H3	0.9558	−0.0213	0.6377	0.0423*	
H4	0.8144	0.1558	0.6120	0.0394*	
H5	0.6741	0.3137	0.5931	0.0567*	
H6	0.5236	0.4784	0.5889	0.0567*	
H7	0.3725	0.4798	0.6688	0.0567*	
H8	0.3747	0.3165	0.7502	0.0567*	
H9	0.5994	−0.1236	0.9532	0.0318*	
H10	0.6134	−0.2983	0.9922	0.0406*	
H11	0.5575	−0.3663	0.8807	0.0449*	
H12	0.4847	−0.2531	0.7346	0.0409*	
H13	0.4076	−0.1374	0.6071	0.0506*	
H14	0.3244	−0.0011	0.4717	0.0643*	
H15	0.3253	0.1676	0.4587	0.0664*	
H16	0.4087	0.1988	0.5790	0.0520*	
H17	−0.1012	0.3495	0.8123	0.0358*	
H18	−0.2825	0.3711	0.8264	0.0398*	
H19	−0.3175	0.2174	0.8697	0.0368*	
H20	−0.1692	0.0450	0.8922	0.0295*	
H21	−0.0217	−0.1096	0.9047	0.0327*	
H22	0.1436	−0.2668	0.9234	0.0403*	
H23	0.3121	−0.2516	0.9094	0.0408*	
H24	0.3108	−0.0816	0.8746	0.0338*	
H25	0.1339	0.3629	0.8916	0.0386*	
H26	0.1370	0.5263	0.8194	0.0452*	
H27	0.1280	0.5747	0.6587	0.0487*	
H28	0.1110	0.4591	0.5736	0.0415*	
H29	0.0643	0.3599	0.5056	0.0376*	
H30	0.0462	0.2307	0.4397	0.0423*	
H31	0.0750	0.0622	0.5338	0.0426*	
H32	0.1158	0.0295	0.6906	0.0374*	
H33	0.0726	0.3639	0.1159	0.0639*	
H34	0.1312	0.3968	0.1895	0.0639*	
H35	0.2046	0.3224	0.1247	0.0639*	
H38	−0.3259	0.4857	0.0557	0.2818*	
H39	−0.3467	0.4287	0.1545	0.2818*	
H40	−0.3557	0.5493	0.1360	0.2818*	
H36	−0.5080	0.6172	0.0737	0.2429*	
H37	−0.5124	0.5115	0.1342	0.2429*	
P1	0.21851 (10)	0.76170 (9)	0.62886 (8)	0.0261 (3)	
F1	0.2049 (3)	0.6869 (3)	0.5677 (3)	0.0447 (8)	
F2	0.2311 (3)	0.8381 (3)	0.6886 (3)	0.0439 (8)	
F3A	0.1258 (4)	0.7458 (4)	0.6902 (3)	0.0543 (13)	0.908 (7)
F4A	0.3037 (4)	0.7831 (4)	0.5619 (3)	0.0524 (13)	0.908 (7)
F5A	0.1200 (4)	0.8654 (3)	0.5615 (3)	0.0521 (12)	0.908 (7)
F6A	0.3127 (5)	0.6621 (4)	0.6928 (4)	0.0727 (17)	0.908 (7)
F5B	0.097 (3)	0.833 (3)	0.623 (3)	0.043 (10)*	0.092 (7)
F3B	0.207 (3)	0.682 (3)	0.721 (2)	0.031 (8)*	0.092 (7)
F4B	0.231 (3)	0.839 (3)	0.553 (2)	0.027 (8)*	0.092 (7)
F6B	0.357 (3)	0.692 (3)	0.637 (3)	0.040 (9)*	0.092 (7)

### Atomic displacement parameters (Å^2^)

**Table:**

	*U*^11^	*U*^22^	*U*^33^	*U*^12^	*U*^13^	*U*^23^
Ru1	0.01871 (17)	0.02358 (18)	0.0339 (2)	−0.01019 (14)	0.00938 (14)	−0.00483 (14)
Ru2	0.01634 (16)	0.02772 (18)	0.02381 (17)	−0.01239 (14)	0.00159 (12)	0.00086 (13)
P2	0.0459 (9)	0.0423 (8)	0.0637 (11)	−0.0248 (7)	0.0133 (8)	−0.0159 (8)
F7	0.145 (5)	0.049 (3)	0.068 (3)	−0.052 (3)	−0.032 (3)	0.005 (2)
F8	0.154 (6)	0.090 (4)	0.102 (4)	−0.094 (4)	0.056 (4)	−0.054 (3)
F9	0.040 (3)	0.090 (5)	0.292 (11)	−0.015 (3)	0.007 (5)	−0.034 (6)
F10	0.058 (3)	0.119 (5)	0.096 (4)	−0.019 (3)	−0.002 (3)	−0.024 (4)
F11	0.153 (6)	0.089 (4)	0.100 (4)	−0.076 (4)	0.077 (4)	−0.045 (3)
F12	0.140 (5)	0.043 (3)	0.064 (3)	−0.033 (3)	0.013 (3)	−0.004 (2)
O1	0.041 (2)	0.0340 (19)	0.037 (2)	−0.0168 (16)	−0.0034 (16)	−0.0046 (15)
O2	0.0253 (16)	0.0343 (18)	0.0317 (17)	−0.0134 (14)	0.0036 (13)	−0.0156 (14)
O3	0.044 (3)	0.057 (3)	0.043 (3)	−0.031 (2)	0.0142 (18)	−0.0288 (19)
O4	0.037 (2)	0.050 (3)	0.0289 (18)	−0.0291 (18)	0.0016 (15)	−0.0044 (16)
N1	0.0219 (18)	0.0305 (19)	0.0186 (17)	−0.0147 (15)	0.0056 (14)	−0.0083 (14)
N2	0.036 (3)	0.025 (2)	0.049 (3)	−0.0135 (18)	0.018 (2)	−0.0062 (18)
N3	0.0173 (17)	0.0268 (19)	0.0288 (19)	−0.0113 (15)	0.0036 (14)	−0.0049 (15)
N4	0.0183 (18)	0.038 (3)	0.036 (3)	−0.0142 (17)	0.0015 (16)	0.0058 (18)
N5	0.0223 (18)	0.0255 (18)	0.0226 (17)	−0.0160 (15)	0.0051 (14)	−0.0079 (14)
N6	0.0223 (18)	0.0267 (18)	0.0220 (18)	−0.0129 (15)	0.0040 (14)	−0.0005 (14)
N7	0.0195 (18)	0.0293 (19)	0.0299 (19)	−0.0146 (15)	0.0014 (15)	−0.0042 (16)
N8	0.0181 (17)	0.0208 (17)	0.0260 (18)	−0.0055 (14)	0.0043 (14)	−0.0023 (14)
N9	0.087 (5)	0.064 (4)	0.063 (4)	−0.046 (4)	−0.003 (4)	−0.010 (4)
C1	0.034 (3)	0.031 (3)	0.034 (3)	−0.015 (2)	0.001 (2)	−0.003 (2)
C2	0.032 (3)	0.032 (3)	0.041 (3)	−0.018 (2)	0.019 (3)	−0.014 (2)
C3	0.022 (3)	0.035 (3)	0.024 (2)	−0.0125 (19)	0.0038 (17)	−0.0075 (18)
C4	0.025 (3)	0.048 (3)	0.027 (3)	−0.011 (3)	0.0040 (19)	−0.013 (2)
C5	0.025 (3)	0.060 (4)	0.032 (3)	−0.024 (3)	0.0116 (19)	−0.020 (3)
C6	0.036 (3)	0.050 (3)	0.026 (3)	−0.029 (3)	0.012 (2)	−0.014 (2)
C7	0.030 (3)	0.036 (3)	0.021 (2)	−0.021 (2)	0.0069 (17)	−0.0101 (18)
C8	0.0556 (17)	0.0331 (13)	0.0547 (16)	−0.0222 (12)	0.0181 (13)	−0.0072 (12)
C9	0.0556 (17)	0.0331 (13)	0.0547 (16)	−0.0222 (12)	0.0181 (13)	−0.0072 (12)
C10	0.0556 (17)	0.0331 (13)	0.0547 (16)	−0.0222 (12)	0.0181 (13)	−0.0072 (12)
C11	0.0556 (17)	0.0331 (13)	0.0547 (16)	−0.0222 (12)	0.0181 (13)	−0.0072 (12)
C12	0.0556 (17)	0.0331 (13)	0.0547 (16)	−0.0222 (12)	0.0181 (13)	−0.0072 (12)
C13	0.023 (2)	0.032 (3)	0.025 (3)	−0.0135 (19)	0.0036 (17)	−0.0052 (18)
C14	0.032 (3)	0.030 (3)	0.032 (3)	−0.009 (2)	0.004 (2)	−0.001 (2)
C15	0.046 (3)	0.032 (3)	0.039 (3)	−0.021 (3)	0.011 (3)	−0.008 (3)
C16	0.039 (3)	0.039 (3)	0.037 (3)	−0.025 (3)	0.009 (3)	−0.016 (3)
C17	0.022 (2)	0.036 (3)	0.028 (3)	−0.0179 (19)	0.0066 (17)	−0.0073 (19)
C18	0.024 (3)	0.049 (3)	0.028 (3)	−0.023 (3)	0.0025 (18)	0.000 (2)
C19	0.036 (3)	0.071 (4)	0.031 (3)	−0.036 (3)	0.002 (3)	−0.008 (3)
C20	0.037 (3)	0.100 (6)	0.030 (3)	−0.041 (4)	−0.004 (3)	−0.005 (3)
C21	0.031 (3)	0.080 (5)	0.040 (4)	−0.025 (3)	−0.006 (3)	0.018 (3)
C22	0.023 (3)	0.054 (4)	0.041 (3)	−0.017 (3)	−0.003 (2)	0.012 (3)
C23	0.021 (2)	0.035 (3)	0.035 (3)	−0.0194 (19)	−0.0010 (18)	0.002 (2)
C24	0.028 (3)	0.027 (3)	0.038 (3)	−0.0140 (19)	0.008 (2)	−0.010 (2)
C25	0.024 (3)	0.031 (3)	0.042 (3)	−0.010 (2)	0.004 (2)	−0.009 (2)
C26	0.020 (3)	0.043 (3)	0.032 (3)	−0.017 (2)	0.0007 (18)	−0.007 (2)
C27	0.025 (3)	0.032 (3)	0.025 (2)	−0.0194 (19)	0.0025 (17)	−0.0076 (18)
C28	0.023 (2)	0.026 (2)	0.0181 (18)	−0.0157 (17)	0.0021 (15)	−0.0063 (16)
C29	0.023 (2)	0.029 (3)	0.0174 (19)	−0.0147 (18)	0.0030 (15)	−0.0045 (16)
C30	0.032 (3)	0.031 (3)	0.025 (3)	−0.020 (2)	0.0048 (18)	−0.0059 (18)
C31	0.044 (3)	0.029 (3)	0.030 (3)	−0.018 (3)	0.007 (2)	−0.0063 (19)
C32	0.032 (3)	0.027 (3)	0.035 (3)	−0.006 (2)	0.005 (2)	−0.006 (2)
C33	0.024 (3)	0.033 (3)	0.026 (3)	−0.0116 (19)	0.0038 (17)	−0.0042 (18)
C34	0.030 (3)	0.035 (3)	0.038 (3)	−0.019 (2)	0.004 (2)	−0.010 (2)
C35	0.038 (3)	0.037 (3)	0.049 (3)	−0.024 (3)	0.006 (3)	−0.016 (3)
C36	0.040 (3)	0.031 (3)	0.057 (4)	−0.024 (3)	0.004 (3)	−0.004 (3)
C37	0.037 (3)	0.031 (3)	0.038 (3)	−0.022 (3)	0.001 (2)	0.002 (2)
C38	0.022 (2)	0.023 (2)	0.031 (3)	−0.0126 (17)	−0.0001 (17)	0.0002 (18)
C39	0.0159 (19)	0.023 (2)	0.029 (3)	−0.0068 (16)	0.0008 (16)	−0.0004 (17)
C40	0.032 (3)	0.027 (3)	0.030 (3)	−0.013 (2)	−0.0042 (19)	0.0031 (19)
C41	0.039 (3)	0.039 (3)	0.027 (3)	−0.018 (3)	0.000 (2)	−0.004 (2)
C42	0.044 (3)	0.034 (3)	0.035 (3)	−0.020 (3)	0.011 (3)	−0.014 (2)
C43	0.037 (3)	0.022 (3)	0.032 (3)	−0.012 (2)	0.010 (2)	−0.0053 (18)
C44	0.046 (4)	0.045 (4)	0.062 (4)	−0.015 (3)	0.000 (3)	−0.010 (3)
C45	0.047 (4)	0.049 (4)	0.054 (4)	−0.024 (3)	0.002 (3)	−0.024 (3)
P1	0.0313 (6)	0.0209 (6)	0.0319 (6)	−0.0144 (5)	0.0094 (5)	−0.0112 (5)
F1	0.060 (2)	0.0445 (18)	0.055 (2)	−0.0377 (17)	0.0234 (17)	−0.0304 (16)
F2	0.063 (3)	0.0435 (18)	0.0424 (18)	−0.0333 (17)	0.0125 (16)	−0.0225 (15)
F3A	0.069 (3)	0.067 (3)	0.059 (3)	−0.051 (3)	0.040 (3)	−0.035 (3)
F4A	0.059 (3)	0.080 (4)	0.055 (3)	−0.055 (3)	0.032 (2)	−0.038 (3)
F5A	0.055 (3)	0.039 (2)	0.047 (3)	−0.0070 (18)	−0.0049 (19)	−0.0069 (17)
F6A	0.081 (4)	0.034 (3)	0.065 (3)	0.010 (3)	−0.022 (3)	−0.010 (2)

### Geometric parameters (Å, º)

**Table:**

Ru1—N1	2.154 (4)	C29—C30	1.387 (8)
Ru1—N2	2.095 (5)	C30—C31	1.380 (6)
Ru1—N3	2.055 (5)	C31—C32	1.381 (9)
Ru1—N4	2.124 (5)	C32—C33	1.374 (9)
Ru1—C1	2.068 (6)	C34—C35	1.385 (9)
Ru1—C2	1.867 (6)	C35—C36	1.378 (9)
Ru2—O1	2.097 (4)	C36—C37	1.382 (10)
Ru2—N5	2.103 (4)	C37—C38	1.392 (8)
Ru2—N6	2.069 (5)	C38—C39	1.469 (8)
Ru2—N7	2.068 (5)	C39—C40	1.385 (7)
Ru2—N8	2.125 (4)	C40—C41	1.375 (10)
Ru2—C23	1.837 (6)	C41—C42	1.378 (8)
P2—F7	1.592 (6)	C42—C43	1.372 (8)
P2—F8	1.567 (8)	C44—C45	1.459 (10)
P2—F9	1.548 (6)	C46—C47	1.37 (4)
P2—F10	1.574 (5)	P1—F1	1.595 (5)
P2—F11	1.592 (6)	P1—F2	1.593 (5)
P2—F12	1.589 (6)	P1—F3A	1.595 (6)
O1—C1	1.269 (9)	P1—F4A	1.588 (6)
O2—C1	1.254 (7)	P1—F5A	1.620 (4)
O3—C2	1.134 (8)	P1—F6A	1.550 (4)
O4—C23	1.159 (7)	P1—F5B	1.49 (4)
O5—O5^i^	0.90 (4)	P1—F3B	1.63 (4)
O5—C46	1.42 (5)	P1—F4B	1.45 (4)
O5—C46^i^	1.68 (5)	P1—F6B	1.68 (4)
N1—C3	1.338 (6)	C3—H1	0.950
N1—C7	1.359 (7)	C4—H2	0.950
N2—C8	1.349 (9)	C5—H3	0.950
N2—C12	1.339 (7)	C6—H4	0.950
N3—C13	1.345 (6)	C9—H5	0.950
N3—C17	1.360 (8)	C10—H6	0.950
N4—C18	1.360 (9)	C11—H7	0.950
N4—C22	1.337 (7)	C12—H8	0.950
N5—C24	1.335 (5)	C13—H9	0.950
N5—C28	1.358 (7)	C14—H10	0.950
N6—C29	1.362 (7)	C15—H11	0.950
N6—C33	1.344 (5)	C16—H12	0.950
N7—C34	1.347 (8)	C19—H13	0.950
N7—C38	1.358 (6)	C20—H14	0.950
N8—C39	1.360 (6)	C21—H15	0.950
N8—C43	1.339 (8)	C22—H16	0.950
N9—C45	1.127 (10)	C24—H17	0.950
C3—C4	1.377 (8)	C25—H18	0.950
C4—C5	1.380 (9)	C26—H19	0.950
C5—C6	1.390 (7)	C27—H20	0.950
C6—C7	1.392 (8)	C30—H21	0.950
C7—C8	1.474 (7)	C31—H22	0.950
C8—C9	1.390 (10)	C32—H23	0.950
C9—C10	1.369 (8)	C33—H24	0.950
C10—C11	1.367 (10)	C34—H25	0.950
C11—C12	1.390 (10)	C35—H26	0.950
C13—C14	1.376 (8)	C36—H27	0.950
C14—C15	1.385 (10)	C37—H28	0.950
C15—C16	1.378 (7)	C40—H29	0.950
C16—C17	1.388 (9)	C41—H30	0.950
C17—C18	1.469 (6)	C42—H31	0.950
C18—C19	1.386 (10)	C43—H32	0.950
C19—C20	1.389 (8)	C44—H33	0.980
C20—C21	1.371 (14)	C44—H34	0.980
C21—C22	1.391 (11)	C44—H35	0.980
C24—C25	1.379 (8)	C46—H36	0.990
C25—C26	1.378 (9)	C46—H37	0.990
C26—C27	1.386 (6)	C47—H38	0.980
C27—C28	1.387 (7)	C47—H39	0.980
C28—C29	1.476 (6)	C47—H40	0.980
			
Ru2···O2	3.126 (4)	O5···H11^ix^	2.7001
O1···N3	3.033 (5)	O5···H11^x^	2.6647
O1···N4	2.905 (6)	O5···H23^ix^	2.9389
O1···C17	3.313 (6)	N1···H14^ii^	3.0383
O1···C18	3.221 (7)	N1···H20^vi^	3.3320
O1···C22	3.582 (7)	N2···H19^vi^	3.1271
O1···C33	3.035 (8)	N3···H35^ii^	3.5993
O1···C38	3.420 (5)	N4···H14^ii^	3.4750
O1···C39	3.398 (5)	N6···H20^vii^	3.4391
O1···C43	3.532 (8)	N9···H17^iv^	3.2739
O2···O3	3.337 (5)	N9···H22^xix^	2.7386
O2···O4	3.234 (6)	N9···H25^xvi^	3.3865
O2···N2	3.489 (7)	N9···H25^iv^	3.4687
O2···N7	2.996 (5)	N9···H26^xvi^	2.8936
O2···C2	2.853 (6)	N9···H33^xi^	2.7344
O2···C12	3.241 (8)	N9···H38^xi^	2.8462
O2···C23	2.818 (7)	C1···H9^v^	3.1803
O2···C34	2.935 (5)	C2···H9^v^	3.2380
O3···C1	3.466 (8)	C2···H19^vi^	2.8467
N1···C5	2.766 (7)	C2···H20^vi^	3.2081
N2···C10	2.772 (8)	C3···H14^ii^	3.1221
N2···C22	3.234 (10)	C3···H20^vi^	3.4620
N3···C3	3.328 (7)	C3···H35^ii^	3.4876
N3···C15	2.779 (8)	C4···H14^ii^	3.2804
N4···C20	2.774 (10)	C4···H21^vi^	3.2908
N5···C26	2.765 (7)	C4···H30^ii^	3.0955
N5···C43	3.565 (7)	C5···H14^ii^	3.3517
N6···C31	2.766 (8)	C5···H30^ii^	3.5026
N6···C43	3.211 (6)	C5···H31^vi^	3.4828
N7···C1	3.220 (6)	C5···H31^ii^	3.1244
N7···C24	3.384 (8)	C5···H32^vi^	3.3832
N7···C36	2.782 (8)	C6···H14^ii^	3.2433
N8···C41	2.761 (7)	C7···H14^ii^	3.0875
C1···C12	3.215 (10)	C7···H19^vi^	3.4555
C1···C23	3.241 (8)	C7···H20^vi^	3.3908
C2···C13	3.106 (9)	C8···H19^vi^	3.1280
C3···C6	2.727 (9)	C10···H6^iii^	3.1333
C4···C7	2.743 (7)	C11···H40^iv^	3.1720
C6···C9	3.011 (7)	C12···H19^vi^	3.5837
C8···C11	2.724 (8)	C12···H40^iv^	3.1106
C9···C12	2.708 (11)	C13···H35^ii^	3.0356
C13···C16	2.709 (9)	C14···H35^ii^	2.7558
C14···C17	2.741 (6)	C14···H38^xiii^	3.3959
C16···C19	2.991 (7)	C14···H40^xiii^	3.4696
C18···C21	2.726 (8)	C14···H36^xiii^	3.0672
C19···C22	2.735 (11)	C15···H35^ii^	3.1086
C20···C42	3.459 (9)	C15···H38^xiii^	3.4932
C21···C41	3.557 (9)	C15···H36^xiii^	3.2982
C23···C29	3.586 (9)	C15···H37^x^	3.2897
C23···C34	3.130 (8)	C18···H15^ii^	3.5567
C24···C27	2.722 (9)	C19···H15^ii^	3.4455
C25···C28	2.735 (6)	C22···H14^ii^	3.4601
C27···C30	3.028 (6)	C23···H9^v^	3.2492
C29···C32	2.744 (6)	C23···H20^vii^	3.2256
C29···C43	3.589 (7)	C23···H21^vii^	2.9737
C30···C33	2.716 (9)	C25···H37^xx^	3.4960
C34···C37	2.728 (8)	C26···H36^xx^	3.4627
C35···C38	2.731 (9)	C28···H21^vii^	3.5995
C37···C40	2.993 (10)	C29···H20^vii^	3.5903
C39···C42	2.734 (9)	C31···H39^x^	3.5636
C40···C43	2.713 (7)	C32···H19^vii^	3.4551
F7···C16^ii^	3.429 (8)	C32···H39^x^	3.3517
F7···C21	3.238 (8)	C34···H34^iv^	3.5179
F7···C44	3.318 (9)	C35···H33^iv^	3.2930
F8···C9^iii^	3.228 (12)	C35···H34^iv^	3.2586
F8···C10^iii^	3.237 (12)	C35···H40^iv^	3.0553
F8···C24^iv^	3.535 (7)	C36···H29^iv^	3.4786
F8···C25^iv^	3.444 (8)	C36···H30^iv^	3.4998
F9···N7^iv^	3.508 (6)	C37···H29^iv^	3.1660
F9···C24^iv^	3.427 (9)	C40···H28^iv^	3.3901
F9···C36^iv^	3.480 (10)	C41···H2^ii^	3.4567
F9···C37^iv^	3.145 (8)	C41···H27^iv^	3.4320
F9···C38^iv^	3.137 (7)	C42···H3^xv^	3.0810
F10···C16^ii^	3.321 (9)	C42···H4^xv^	3.4628
F10···F6B^iii^	3.16 (4)	C43···H3^xv^	3.1052
F11···C21	3.361 (10)	C44···H1^ii^	3.1872
F11···C22	3.371 (7)	C44···H26^iv^	3.4355
F11···C37	3.205 (9)	C45···H10^ii^	3.5764
F11···C40	3.180 (10)	C45···H17^iv^	3.3105
F12···C15^ii^	3.282 (8)	C45···H25^xvi^	3.2466
F12···C16^ii^	3.380 (7)	C45···H25^iv^	3.3825
F12···C24^iv^	3.303 (6)	C45···H26^xvi^	3.5255
F12···C25^iv^	3.130 (7)	C45···H33^xi^	3.3502
F12···C44	3.370 (11)	C45···H38^xi^	3.3547
F12···C45	3.527 (10)	C46···H10^ix^	3.0876
O1···O3^v^	3.582 (5)	C46···H11^ix^	3.1215
O2···C13^v^	3.106 (6)	C46···H11^x^	2.9422
O2···C14^v^	3.264 (8)	C46···H18^xx^	3.3131
O3···O1^v^	3.582 (5)	C46···H19^xx^	3.5982
O3···O4^v^	3.319 (5)	C46···H23^ix^	3.5883
O3···C1^v^	3.585 (7)	C47···H7^iv^	3.4115
O3···C13^v^	3.098 (7)	C47···H10^ix^	2.9185
O3···C14^v^	3.436 (7)	C47···H11^ix^	3.4425
O3···C23^v^	3.344 (5)	C47···H22^x^	3.4990
O3···C26^vi^	3.393 (8)	C47···H23^x^	3.2727
O3···C27^vi^	3.533 (7)	C47···H26^iv^	3.1371
O3···C33^v^	3.094 (8)	H1···F7^ii^	3.3530
O4···O3^v^	3.319 (5)	H1···O4^v^	3.0466
O4···C2^v^	3.499 (5)	H1···C44^ii^	3.1872
O4···C3^v^	3.402 (6)	H1···H33^ii^	3.2088
O4···C13^v^	3.373 (7)	H1···H34^ii^	3.2673
O4···C30^vii^	3.473 (8)	H1···H35^ii^	2.5949
O4···C44^viii^	3.520 (10)	H2···C41^ii^	3.4567
O5···C15^ix^	3.42 (4)	H2···H21^vi^	3.3163
O5···C15^x^	3.52 (3)	H2···H30^ii^	2.6082
N7···F9^iv^	3.508 (6)	H2···H33^ii^	3.2848
N9···C44^xi^	3.520 (11)	H2···H34^ii^	3.5553
N9···C45^xi^	3.514 (12)	H2···F3A^xii^	2.5611
C1···O3^v^	3.585 (7)	H2···F5B^xii^	2.7228
C2···O4^v^	3.499 (5)	H2···F3B^xii^	3.5049
C2···C26^vi^	3.581 (9)	H3···C42^vi^	3.0810
C3···O4^v^	3.402 (6)	H3···C43^vi^	3.1052
C4···F3A^xii^	3.300 (7)	H3···H30^ii^	3.3859
C4···F5B^xii^	3.17 (4)	H3···H31^vi^	2.6516
C5···F3A^xii^	3.523 (6)	H3···H31^ii^	2.8164
C5···F5A^xii^	3.390 (7)	H3···H32^vi^	2.6823
C5···F5A^iii^	3.452 (7)	H3···P1^xii^	3.5755
C5···F5B^xii^	2.91 (4)	H3···F2^xii^	3.3537
C5···F4B^iii^	3.44 (3)	H3···F3A^xii^	3.0177
C6···C27^vi^	3.565 (8)	H3···F5A^xii^	2.5432
C6···F4A^iii^	3.188 (6)	H3···F5A^iii^	3.1651
C6···F5A^iii^	3.255 (7)	H3···F5B^xii^	2.1793
C6···F4B^iii^	2.88 (3)	H3···F4B^iii^	3.5285
C7···C26^vi^	3.536 (9)	H4···C42^vi^	3.4628
C7···C27^vi^	3.507 (8)	H4···H31^vi^	3.4594
C7···F4A^iii^	3.570 (6)	H4···P1^iii^	3.5058
C7···F4B^iii^	3.58 (3)	H4···F1^iii^	3.0143
C8···C26^vi^	3.431 (10)	H4···F4A^iii^	2.8145
C9···F8^iii^	3.228 (12)	H4···F5A^iii^	2.7736
C9···F4A^iii^	3.356 (9)	H4···F4B^iii^	2.5255
C10···F8^iii^	3.237 (12)	H5···F8^iii^	2.7661
C11···F6A	3.270 (8)	H5···F1^iii^	2.8878
C11···F6B	3.52 (4)	H5···F4A^iii^	2.8851
C13···O2^v^	3.106 (6)	H5···F4B^iii^	3.2710
C13···O3^v^	3.098 (7)	H5···F6B^iii^	3.5009
C13···O4^v^	3.373 (7)	H6···F8^iii^	2.8011
C14···O2^v^	3.264 (8)	H6···F10^iii^	3.0783
C14···O3^v^	3.436 (7)	H6···C10^iii^	3.1333
C15···F12^ii^	3.282 (8)	H6···H6^iii^	2.6160
C15···O5^xiii^	3.42 (4)	H6···F6A	3.5475
C15···O5^x^	3.52 (3)	H6···F6B	3.1287
C16···F7^ii^	3.429 (8)	H7···C47^iv^	3.4115
C16···F10^ii^	3.321 (9)	H7···H39^iv^	3.1383
C16···F12^ii^	3.380 (7)	H7···H40^iv^	2.8800
C16···F6B^xiv^	3.58 (5)	H7···H37^iv^	3.5453
C19···C21^ii^	3.449 (9)	H7···P1	3.5139
C19···F2^xiv^	3.268 (9)	H7···F1	2.8959
C19···F4A^xiv^	3.077 (11)	H7···F6A	2.4430
C19···F4B^xiv^	3.25 (5)	H7···F3B	3.0365
C20···C21^ii^	3.529 (9)	H7···F6B	2.8279
C20···C22^ii^	3.517 (8)	H8···H40^iv^	2.7360
C20···F4A^xiv^	3.500 (12)	H9···O2^v^	2.3875
C20···F4B^xiv^	3.44 (5)	H9···O3^v^	2.9315
C21···F7	3.238 (8)	H9···O4^v^	2.8268
C21···F11	3.361 (10)	H9···C1^v^	3.1803
C21···C19^ii^	3.449 (9)	H9···C2^v^	3.2380
C21···C20^ii^	3.529 (9)	H9···C23^v^	3.2492
C22···F11	3.371 (7)	H9···H35^ii^	3.3512
C22···C20^ii^	3.517 (8)	H10···O2^v^	2.7446
C23···O3^v^	3.344 (5)	H10···O3^v^	3.5142
C24···F8^iv^	3.535 (7)	H10···C45^ii^	3.5764
C24···F9^iv^	3.427 (9)	H10···C46^xiii^	3.0876
C24···F12^iv^	3.303 (6)	H10···C47^xiii^	2.9185
C25···F8^iv^	3.444 (8)	H10···H25^v^	3.4641
C25···F12^iv^	3.130 (7)	H10···H35^ii^	2.9290
C26···O3^xv^	3.393 (8)	H10···H38^xiii^	2.7206
C26···C2^xv^	3.581 (9)	H10···H40^xiii^	2.5725
C26···C7^xv^	3.536 (9)	H10···H36^xiii^	2.5380
C26···C8^xv^	3.431 (10)	H11···F12^ii^	2.9873
C26···C32^vii^	3.480 (8)	H11···O5^xiii^	2.7001
C27···O3^xv^	3.533 (7)	H11···O5^x^	2.6647
C27···C6^xv^	3.565 (8)	H11···C46^xiii^	3.1215
C27···C7^xv^	3.507 (8)	H11···C46^x^	2.9422
C28···C30^vii^	3.518 (7)	H11···C47^xiii^	3.4425
C30···O4^vii^	3.473 (8)	H11···H35^ii^	3.4665
C30···C28^vii^	3.518 (7)	H11···H38^xiii^	2.9225
C31···F2^xiv^	3.504 (7)	H11···H39^x^	3.3439
C31···F3A^xiv^	3.583 (8)	H11···H36^xiii^	3.0003
C31···F3B^xiv^	3.52 (4)	H11···H37^x^	2.3990
C32···C26^vii^	3.480 (8)	H12···F7^ii^	3.3539
C32···F2^xiv^	3.123 (7)	H12···F10^ii^	2.6649
C33···O3^v^	3.094 (8)	H12···F12^ii^	3.1626
C33···F2^xiv^	3.301 (7)	H12···H37^x^	3.5851
C35···F3B	3.32 (5)	H12···F2^xiv^	3.0908
C36···F9^iv^	3.480 (10)	H12···F4A^xiv^	3.3497
C36···F1	3.293 (8)	H12···F6A^xiv^	3.1062
C36···F3A	3.321 (9)	H12···F6B^xiv^	2.6744
C36···F3B	3.16 (5)	H13···F10^ii^	3.0738
C37···F9^iv^	3.145 (8)	H13···H15^ii^	3.5622
C37···F11	3.205 (9)	H13···P1^xiv^	3.3459
C38···F9^iv^	3.137 (7)	H13···F2^xiv^	2.7553
C40···F11	3.180 (10)	H13···F4A^xiv^	2.2874
C41···F1^iv^	3.342 (7)	H13···F6A^xiv^	3.5324
C41···F5A^iv^	3.328 (9)	H13···F4B^xiv^	2.6564
C41···F5B^iv^	3.24 (5)	H13···F6B^xiv^	2.6781
C42···F5A^xiv^	3.320 (8)	H14···N1^ii^	3.0383
C42···F5A^iv^	3.122 (8)	H14···N4^ii^	3.4750
C42···F5B^iv^	3.37 (4)	H14···C3^ii^	3.1221
C42···F4B^xiv^	3.57 (4)	H14···C4^ii^	3.2804
C43···F2^xiv^	3.223 (6)	H14···C5^ii^	3.3517
C44···F7	3.318 (9)	H14···C6^ii^	3.2433
C44···F12	3.370 (11)	H14···C7^ii^	3.0875
C44···O4^xvi^	3.520 (10)	H14···C22^ii^	3.4601
C44···N9^xi^	3.520 (11)	H14···F4A^xiv^	3.1493
C45···F12	3.527 (10)	H14···F4B^xiv^	3.0212
C45···N9^xi^	3.514 (12)	H15···P2	3.2966
C45···C45^xi^	3.479 (11)	H15···F7	2.2978
C47···F6A^iv^	3.18 (3)	H15···F10	3.0752
C47···F3B^iv^	3.19 (4)	H15···F11	2.7754
F1···C36	3.293 (8)	H15···C18^ii^	3.5567
F1···C41^iv^	3.342 (7)	H15···C19^ii^	3.4455
F2···C19^xvii^	3.268 (9)	H15···H13^ii^	3.5622
F2···C31^xvii^	3.504 (7)	H16···F10	3.1583
F2···C32^xvii^	3.123 (7)	H16···F11	2.7851
F2···C33^xvii^	3.301 (7)	H17···P2^iv^	3.4826
F2···C43^xvii^	3.223 (6)	H17···F8^iv^	3.2522
F3A···C4^xviii^	3.300 (7)	H17···F9^iv^	2.6421
F3A···C5^xviii^	3.523 (6)	H17···F12^iv^	2.7745
F3A···C31^xvii^	3.583 (8)	H17···N9^iv^	3.2739
F3A···C36	3.321 (9)	H17···C45^iv^	3.3105
F4A···C6^iii^	3.188 (6)	H17···H34^iv^	3.3965
F4A···C7^iii^	3.570 (6)	H18···F8^iv^	3.1015
F4A···C9^iii^	3.356 (9)	H18···F12^iv^	2.4421
F4A···C19^xvii^	3.077 (11)	H18···C46^xx^	3.3131
F4A···C20^xvii^	3.500 (12)	H18···H36^xx^	3.1190
F5A···C5^xviii^	3.390 (7)	H18···H37^xx^	2.6773
F5A···C5^iii^	3.452 (7)	H19···O3^xv^	2.7118
F5A···C6^iii^	3.255 (7)	H19···N2^xv^	3.1271
F5A···C41^iv^	3.328 (9)	H19···C2^xv^	2.8467
F5A···C42^xvii^	3.320 (8)	H19···C7^xv^	3.4555
F5A···C42^iv^	3.122 (8)	H19···C8^xv^	3.1280
F6A···C11	3.270 (8)	H19···C12^xv^	3.5837
F6A···C47^iv^	3.18 (3)	H19···C32^vii^	3.4551
F5B···C4^xviii^	3.17 (4)	H19···C46^xx^	3.5982
F5B···C5^xviii^	2.91 (4)	H19···H23^vii^	3.4466
F5B···C41^iv^	3.24 (5)	H19···H36^xx^	2.7967
F5B···C42^iv^	3.37 (4)	H19···H37^xx^	3.4997
F3B···C31^xvii^	3.52 (4)	H20···O3^xv^	3.0065
F3B···C35	3.32 (5)	H20···O4^vii^	2.9118
F3B···C36	3.16 (5)	H20···N1^xv^	3.3320
F3B···C47^iv^	3.19 (4)	H20···N6^vii^	3.4391
F4B···C5^iii^	3.44 (3)	H20···C2^xv^	3.2081
F4B···C6^iii^	2.88 (3)	H20···C3^xv^	3.4620
F4B···C7^iii^	3.58 (3)	H20···C7^xv^	3.3908
F4B···C19^xvii^	3.25 (5)	H20···C23^vii^	3.2256
F4B···C20^xvii^	3.44 (5)	H20···C29^vii^	3.5903
F4B···C42^xvii^	3.57 (4)	H21···O4^vii^	2.5582
F6B···F10^iii^	3.16 (4)	H21···C4^xv^	3.2908
F6B···C11	3.52 (4)	H21···C23^vii^	2.9737
F6B···C16^xvii^	3.58 (5)	H21···C28^vii^	3.5995
Ru1···H1	3.1853	H21···H2^xv^	3.3163
Ru1···H8	3.1067	H22···N9^xxi^	2.7386
Ru1···H9	3.0948	H22···C47^x^	3.4990
Ru1···H16	3.2004	H22···H38^x^	2.9583
Ru2···H17	3.1503	H22···H39^x^	3.1323
Ru2···H24	3.1038	H22···F3A^xiv^	3.4596
Ru2···H25	3.1285	H22···F3B^xiv^	3.2863
Ru2···H32	3.1935	H23···O5^xiii^	2.9389
O1···H8	3.4853	H23···C46^xiii^	3.5883
O1···H24	2.4673	H23···C47^x^	3.2727
O2···H8	2.4898	H23···H19^vii^	3.4466
O2···H25	2.8237	H23···H38^x^	3.1272
O3···H9	2.8647	H23···H39^x^	2.7093
O4···H25	2.9473	H23···H36^xiii^	3.0941
O5···H38	2.6415	H23···F2^xiv^	3.2779
O5···H38^i^	2.1837	H24···O3^v^	2.6499
O5···H39	2.9907	H24···F2^xiv^	3.5383
O5···H39^i^	2.6746	H25···N9^viii^	3.3865
O5···H40	3.2324	H25···N9^iv^	3.4687
O5···H40^i^	3.1752	H25···C45^viii^	3.2466
N1···H2	3.2328	H25···C45^iv^	3.3825
N1···H4	3.2497	H25···H10^v^	3.4641
N2···H5	3.2384	H25···H33^viii^	3.4515
N2···H7	3.2411	H25···H35^viii^	3.4775
N2···H16	2.6972	H26···N9^viii^	2.8936
N3···H1	2.7881	H26···C44^iv^	3.4355
N3···H10	3.2402	H26···C45^viii^	3.5255
N3···H12	3.2413	H26···C47^iv^	3.1371
N3···H24	3.0647	H26···H33^iv^	2.8514
N4···H13	3.2535	H26···H34^iv^	3.2659
N4···H15	3.2337	H26···H38^iv^	3.0667
N5···H18	3.2322	H26···H39^iv^	3.1507
N5···H20	3.2475	H26···H40^iv^	2.6812
N5···H32	3.5577	H26···F3A	3.1953
N6···H21	3.2384	H26···F3B	2.8322
N6···H23	3.2324	H27···C41^iv^	3.4320
N6···H32	2.6659	H27···H29^iv^	3.2500
N7···H8	3.4180	H27···H30^iv^	2.8246
N7···H17	2.8484	H27···P1	3.2570
N7···H26	3.2456	H27···F1	2.4004
N7···H28	3.2566	H27···F3A	2.5260
N8···H29	3.2420	H27···F6A	3.2554
N8···H31	3.2296	H27···F5B	3.3770
N9···H33	3.0505	H27···F3B	2.4780
N9···H34	3.0583	H28···F9^iv^	3.4526
N9···H35	3.0557	H28···F11	2.4938
C1···H8	2.6430	H28···C40^iv^	3.3901
C1···H24	3.3281	H28···H28^iv^	3.3225
C2···H8	3.5881	H28···H29^iv^	2.6551
C2···H9	2.5908	H29···F7	3.5892
C3···H3	3.2359	H29···F9	3.2192
C4···H4	3.2533	H29···F11	2.6412
C5···H1	3.2264	H29···C36^iv^	3.4786
C6···H2	3.2554	H29···C37^iv^	3.1660
C6···H5	2.7253	H29···H27^iv^	3.2500
C7···H1	3.1803	H29···H28^iv^	2.6551
C7···H3	3.2600	H29···H29^iv^	3.5280
C7···H5	2.7072	H30···C4^ii^	3.0955
C8···H4	2.7235	H30···C5^ii^	3.5026
C8···H6	3.2496	H30···C36^iv^	3.4998
C8···H8	3.1657	H30···H2^ii^	2.6082
C8···H16	3.1412	H30···H3^ii^	3.3859
C9···H4	2.7348	H30···H27^iv^	2.8246
C9···H7	3.2221	H30···F1^iv^	3.0422
C10···H8	3.2280	H30···F3A^iv^	2.8743
C11···H5	3.2195	H30···F5A^iv^	3.0320
C12···H6	3.2416	H30···F5B^iv^	2.6662
C12···H16	3.0038	H31···C5^xv^	3.4828
C13···H1	3.1363	H31···C5^ii^	3.1244
C13···H11	3.2419	H31···H3^xv^	2.6516
C13···H24	3.2564	H31···H3^ii^	2.8164
C14···H12	3.2366	H31···H4^xv^	3.4594
C14···H24	3.4136	H31···H31^x^	3.4258
C15···H9	3.2300	H31···F2^xiv^	3.3095
C15···H23	3.0769	H31···F5A^xiv^	2.4978
C15···H24	3.3647	H31···F5A^iv^	2.6496
C16···H10	3.2397	H31···F5B^xiv^	3.0853
C16···H13	2.7031	H31···F5B^iv^	2.9450
C16···H23	3.3294	H31···F4B^xiv^	2.8906
C16···H24	3.1323	H32···C5^xv^	3.3832
C17···H1	3.2463	H32···H3^xv^	2.6823
C17···H9	3.1785	H32···F2^xiv^	2.4759
C17···H11	3.2610	H32···F5A^xiv^	3.2900
C17···H13	2.6914	H32···F5B^xiv^	3.2309
C17···H24	2.9648	H32···F4B^xiv^	3.5552
C18···H12	2.7123	H33···O4^xvi^	3.1173
C18···H14	3.2526	H33···N9^xi^	2.7344
C18···H16	3.1801	H33···C35^iv^	3.2930
C19···H12	2.7062	H33···C45^xi^	3.3502
C19···H15	3.2433	H33···H1^ii^	3.2088
C20···H16	3.2392	H33···H2^ii^	3.2848
C20···H31	3.5430	H33···H25^xvi^	3.4515
C21···H13	3.2429	H33···H26^iv^	2.8514
C22···H14	3.2504	H34···P2	3.2603
C23···H25	2.6435	H34···F7	2.5425
C24···H19	3.2362	H34···F9	2.6887
C25···H20	3.2479	H34···F12	2.7654
C26···H17	3.2260	H34···C34^iv^	3.5179
C27···H18	3.2498	H34···C35^iv^	3.2586
C27···H21	2.7501	H34···H1^ii^	3.2673
C28···H17	3.1741	H34···H2^ii^	3.5553
C28···H19	3.2517	H34···H17^iv^	3.3965
C28···H21	2.7241	H34···H26^iv^	3.2659
C28···H32	3.4688	H35···F7	3.2461
C29···H20	2.7160	H35···F12	3.4236
C29···H22	3.2586	H35···O4^xvi^	3.0350
C29···H24	3.1884	H35···N3^ii^	3.5993
C29···H32	2.9649	H35···C3^ii^	3.4876
C30···H20	2.7524	H35···C13^ii^	3.0356
C30···H23	3.2412	H35···C14^ii^	2.7558
C30···H32	3.5977	H35···C15^ii^	3.1086
C31···H24	3.2281	H35···H1^ii^	2.5949
C32···H21	3.2384	H35···H9^ii^	3.3512
C33···H22	3.2375	H35···H10^ii^	2.9290
C33···H32	3.0651	H35···H11^ii^	3.4665
C34···H8	3.2774	H35···H25^xvi^	3.4775
C34···H17	3.3020	H38···N9^xi^	2.8462
C34···H27	3.2461	H38···C14^ix^	3.3959
C35···H8	3.2068	H38···C15^ix^	3.4932
C35···H28	3.2437	H38···C45^xi^	3.3547
C36···H7	3.1660	H38···H10^ix^	2.7206
C36···H8	3.2563	H38···H11^ix^	2.9225
C36···H25	3.2350	H38···H22^x^	2.9583
C37···H8	3.3937	H38···H23^x^	3.1272
C37···H26	3.2439	H38···H26^iv^	3.0667
C37···H29	2.7124	H38···F3B^iv^	3.5630
C38···H8	3.4796	H39···C31^x^	3.5636
C38···H17	3.3855	H39···C32^x^	3.3517
C38···H25	3.1763	H39···H7^iv^	3.1383
C38···H27	3.2542	H39···H11^x^	3.3439
C38···H29	2.7086	H39···H22^x^	3.1323
C39···H28	2.6966	H39···H23^x^	2.7093
C39···H30	3.2468	H39···H26^iv^	3.1507
C39···H32	3.1771	H39···F3A^iv^	3.4081
C40···H15	3.4435	H39···F6A^iv^	2.3027
C40···H28	2.7117	H39···F3B^iv^	2.3891
C40···H31	3.2393	H39···F6B^iv^	3.2370
C41···H15	3.3523	H40···O2^iv^	3.5771
C41···H32	3.2208	H40···C11^iv^	3.1720
C42···H14	3.3822	H40···C12^iv^	3.1106
C42···H29	3.2375	H40···C14^ix^	3.4696
C43···H30	3.2302	H40···C35^iv^	3.0553
C46···H38^i^	3.4694	H40···H7^iv^	2.8800
C46···H36^i^	3.5424	H40···H8^iv^	2.7360
C46···H37^i^	3.4407	H40···H10^ix^	2.5725
H1···H2	2.3156	H40···H26^iv^	2.6812
H1···H9	3.3764	H40···F6A^iv^	3.3050
H2···H3	2.3429	H40···F3B^iv^	3.2607
H3···H4	2.3517	H36···C14^ix^	3.0672
H4···H5	2.1766	H36···C15^ix^	3.2982
H5···H6	2.3275	H36···C26^xx^	3.4627
H6···H7	2.3302	H36···H10^ix^	2.5380
H7···H8	2.3301	H36···H11^ix^	3.0003
H7···H27	2.9490	H36···H18^xx^	3.1190
H8···H16	3.2686	H36···H19^xx^	2.7967
H9···H10	2.3117	H36···H23^ix^	3.0941
H10···H11	2.3524	H37···F12^xv^	3.4898
H11···H12	2.3354	H37···C15^x^	3.2897
H11···H23	3.0584	H37···C25^xx^	3.4960
H12···H13	2.1448	H37···H7^iv^	3.5453
H12···H23	3.4705	H37···H11^x^	2.3990
H13···H14	2.3536	H37···H12^x^	3.5851
H14···H15	2.3305	H37···H18^xx^	2.6773
H14···H31	3.2474	H37···H19^xx^	3.4997
H15···H16	2.3310	H37···F6A^iv^	3.4063
H15···H29	3.5785	P1···H3^xviii^	3.5755
H15···H30	3.4404	P1···H4^iii^	3.5058
H17···H18	2.3184	P1···H7	3.5139
H17···H25	3.4453	P1···H13^xvii^	3.3459
H18···H19	2.3412	P1···H27	3.2570
H19···H20	2.3471	F1···H4^iii^	3.0143
H20···H21	2.2030	F1···H5^iii^	2.8878
H21···H22	2.3366	F1···H7	2.8959
H22···H23	2.3447	F1···H27	2.4004
H23···H24	2.3130	F1···H30^iv^	3.0422
H24···H32	3.4018	F2···H3^xviii^	3.3537
H25···H26	2.3230	F2···H12^xvii^	3.0908
H26···H27	2.3397	F2···H13^xvii^	2.7553
H27···H28	2.3445	F2···H23^xvii^	3.2779
H28···H29	2.1665	F2···H24^xvii^	3.5383
H29···H30	2.3322	F2···H31^xvii^	3.3095
H30···H31	2.3418	F2···H32^xvii^	2.4759
H31···H32	2.3108	F3A···H2^xviii^	2.5611
H38···H36	2.4139	F3A···H3^xviii^	3.0177
H38···H37	2.6624	F3A···H22^xvii^	3.4596
H38···H37^i^	3.5570	F3A···H26	3.1953
H39···H36	2.6333	F3A···H27	2.5260
H39···H37	2.0062	F3A···H30^iv^	2.8743
H40···H36	1.9767	F3A···H39^iv^	3.4081
H40···H37	2.3572	F4A···H4^iii^	2.8145
P2···H15	3.2966	F4A···H5^iii^	2.8851
P2···H17^iv^	3.4826	F4A···H12^xvii^	3.3497
P2···H34	3.2603	F4A···H13^xvii^	2.2874
F7···H1^ii^	3.3530	F4A···H14^xvii^	3.1493
F7···H12^ii^	3.3539	F5A···H3^xviii^	2.5432
F7···H15	2.2978	F5A···H3^iii^	3.1651
F7···H29	3.5892	F5A···H4^iii^	2.7736
F7···H34	2.5425	F5A···H30^iv^	3.0320
F7···H35	3.2461	F5A···H31^xvii^	2.4978
F8···H5^iii^	2.7661	F5A···H31^iv^	2.6496
F8···H6^iii^	2.8011	F5A···H32^xvii^	3.2900
F8···H17^iv^	3.2522	F6A···H6	3.5475
F8···H18^iv^	3.1015	F6A···H7	2.4430
F9···H17^iv^	2.6421	F6A···H12^xvii^	3.1062
F9···H28^iv^	3.4526	F6A···H13^xvii^	3.5324
F9···H29	3.2192	F6A···H27	3.2554
F9···H34	2.6887	F6A···H39^iv^	2.3027
F10···H6^iii^	3.0783	F6A···H40^iv^	3.3050
F10···H12^ii^	2.6649	F6A···H37^iv^	3.4063
F10···H13^ii^	3.0738	F5B···H2^xviii^	2.7228
F10···H15	3.0752	F5B···H3^xviii^	2.1793
F10···H16	3.1583	F5B···H27	3.3770
F11···H15	2.7754	F5B···H30^iv^	2.6662
F11···H16	2.7851	F5B···H31^xvii^	3.0853
F11···H28	2.4938	F5B···H31^iv^	2.9450
F11···H29	2.6412	F5B···H32^xvii^	3.2309
F12···H11^ii^	2.9873	F3B···H2^xviii^	3.5049
F12···H12^ii^	3.1626	F3B···H7	3.0365
F12···H17^iv^	2.7745	F3B···H22^xvii^	3.2863
F12···H18^iv^	2.4421	F3B···H26	2.8322
F12···H34	2.7654	F3B···H27	2.4780
F12···H35	3.4236	F3B···H38^iv^	3.5630
F12···H37^vi^	3.4898	F3B···H39^iv^	2.3891
O2···H9^v^	2.3875	F3B···H40^iv^	3.2607
O2···H10^v^	2.7446	F4B···H3^iii^	3.5285
O2···H40^iv^	3.5771	F4B···H4^iii^	2.5255
O3···H9^v^	2.9315	F4B···H5^iii^	3.2710
O3···H10^v^	3.5142	F4B···H13^xvii^	2.6564
O3···H19^vi^	2.7118	F4B···H14^xvii^	3.0212
O3···H20^vi^	3.0065	F4B···H31^xvii^	2.8906
O3···H24^v^	2.6499	F4B···H32^xvii^	3.5552
O4···H1^v^	3.0466	F6B···H5^iii^	3.5009
O4···H9^v^	2.8268	F6B···H6	3.1287
O4···H20^vii^	2.9118	F6B···H7	2.8279
O4···H21^vii^	2.5582	F6B···H12^xvii^	2.6744
O4···H33^viii^	3.1173	F6B···H13^xvii^	2.6781
O4···H35^viii^	3.0350	F6B···H39^iv^	3.2370
			
N1—Ru1—N2	76.63 (16)	C37—C38—C39	122.5 (5)
N1—Ru1—N3	96.64 (15)	N8—C39—C38	115.5 (4)
N1—Ru1—N4	89.32 (16)	N8—C39—C40	120.8 (5)
N1—Ru1—C1	172.8 (2)	C38—C39—C40	123.7 (5)
N1—Ru1—C2	94.8 (2)	C39—C40—C41	119.6 (5)
N2—Ru1—N3	169.50 (18)	C40—C41—C42	119.4 (5)
N2—Ru1—N4	93.4 (2)	C41—C42—C43	118.8 (6)
N2—Ru1—C1	96.3 (2)	N8—C43—C42	122.7 (5)
N2—Ru1—C2	92.9 (3)	N9—C45—C44	179.2 (10)
N3—Ru1—N4	78.34 (17)	O5—C46—O5^i^	32.5 (14)
N3—Ru1—C1	90.5 (2)	O5—C46—C47	130 (3)
N3—Ru1—C2	95.7 (2)	O5^i^—C46—C47	98.0 (19)
N4—Ru1—C1	92.3 (2)	F1—P1—F2	179.08 (16)
N4—Ru1—C2	173.2 (3)	F1—P1—F3A	89.7 (3)
C1—Ru1—C2	84.3 (3)	F1—P1—F4A	88.9 (3)
O1—Ru2—N5	164.54 (18)	F1—P1—F5A	88.9 (2)
O1—Ru2—N6	90.68 (16)	F1—P1—F6A	90.8 (3)
O1—Ru2—N7	89.50 (15)	F1—P1—F5B	95.1 (19)
O1—Ru2—N8	81.85 (15)	F1—P1—F3B	90.1 (15)
O1—Ru2—C23	97.35 (18)	F1—P1—F4B	95.9 (15)
N5—Ru2—N6	78.35 (15)	F1—P1—F6B	90.3 (16)
N5—Ru2—N7	99.72 (15)	F2—P1—F3A	90.6 (3)
N5—Ru2—N8	87.88 (15)	F2—P1—F4A	90.7 (3)
N5—Ru2—C23	94.02 (19)	F2—P1—F5A	90.3 (2)
N6—Ru2—N7	171.39 (17)	F2—P1—F6A	90.1 (3)
N6—Ru2—N8	93.36 (16)	F2—P1—F5B	84.4 (19)
N6—Ru2—C23	92.5 (2)	F2—P1—F3B	90.7 (15)
N7—Ru2—N8	78.14 (17)	F2—P1—F4B	83.3 (15)
N7—Ru2—C23	96.0 (2)	F2—P1—F6B	90.1 (16)
N8—Ru2—C23	174.1 (3)	F3A—P1—F4A	175.8 (2)
F7—P2—F8	177.8 (4)	F3A—P1—F5A	88.1 (3)
F7—P2—F9	90.5 (4)	F3A—P1—F6A	92.0 (3)
F7—P2—F10	87.2 (4)	F3A—P1—F6B	132.6 (13)
F7—P2—F11	90.6 (4)	F4A—P1—F5A	87.9 (3)
F7—P2—F12	87.8 (3)	F4A—P1—F6A	92.1 (3)
F8—P2—F9	89.6 (4)	F5A—P1—F6A	179.6 (3)
F8—P2—F10	92.7 (4)	F5B—P1—F3B	89.4 (19)
F8—P2—F11	91.6 (4)	F5B—P1—F4B	91 (2)
F8—P2—F12	90.0 (3)	F5B—P1—F6B	174 (3)
F9—P2—F10	177.6 (5)	F3B—P1—F4B	174 (2)
F9—P2—F11	93.4 (5)	F3B—P1—F6B	93.0 (17)
F9—P2—F12	89.0 (5)	F4B—P1—F6B	86.5 (18)
F10—P2—F11	87.4 (4)	N1—C3—H1	118.658
F10—P2—F12	90.2 (3)	C4—C3—H1	118.656
F11—P2—F12	177.2 (3)	C3—C4—H2	120.578
Ru2—O1—C1	121.5 (4)	C5—C4—H2	120.589
O5^i^—O5—C46	90 (4)	C4—C5—H3	120.305
O5^i^—O5—C46^i^	57 (3)	C6—C5—H3	120.297
C46—O5—C46^i^	147 (2)	C5—C6—H4	120.497
Ru1—N1—C3	125.6 (4)	C7—C6—H4	120.492
Ru1—N1—C7	115.0 (3)	C8—C9—H5	120.094
C3—N1—C7	119.1 (4)	C10—C9—H5	120.106
Ru1—N2—C8	117.5 (4)	C9—C10—H6	120.464
Ru1—N2—C12	124.1 (5)	C11—C10—H6	120.465
C8—N2—C12	118.4 (6)	C10—C11—H7	120.465
Ru1—N3—C13	125.0 (4)	C12—C11—H7	120.472
Ru1—N3—C17	116.6 (3)	N2—C12—H8	118.827
C13—N3—C17	118.4 (5)	C11—C12—H8	118.822
Ru1—N4—C18	114.0 (3)	N3—C13—H9	118.490
Ru1—N4—C22	127.1 (5)	C14—C13—H9	118.502
C18—N4—C22	118.8 (6)	C13—C14—H10	120.535
Ru2—N5—C24	126.1 (4)	C15—C14—H10	120.540
Ru2—N5—C28	115.1 (3)	C14—C15—H11	120.700
C24—N5—C28	118.8 (5)	C16—C15—H11	120.712
Ru2—N6—C29	116.0 (3)	C15—C16—H12	119.836
Ru2—N6—C33	124.7 (4)	C17—C16—H12	119.836
C29—N6—C33	119.2 (5)	C18—C19—H13	120.587
Ru2—N7—C34	125.5 (3)	C20—C19—H13	120.572
Ru2—N7—C38	116.1 (4)	C19—C20—H14	120.429
C34—N7—C38	118.0 (5)	C21—C20—H14	120.418
Ru2—N8—C39	114.3 (4)	C20—C21—H15	120.221
Ru2—N8—C43	127.0 (3)	C22—C21—H15	120.200
C39—N8—C43	118.7 (4)	N4—C22—H16	119.096
Ru1—C1—O1	112.6 (4)	C21—C22—H16	119.087
Ru1—C1—O2	125.0 (5)	N5—C24—H17	118.659
O1—C1—O2	122.4 (5)	C25—C24—H17	118.648
Ru1—C2—O3	178.3 (6)	C24—C25—H18	120.615
N1—C3—C4	122.7 (5)	C26—C25—H18	120.613
C3—C4—C5	118.8 (5)	C25—C26—H19	120.302
C4—C5—C6	119.4 (5)	C27—C26—H19	120.297
C5—C6—C7	119.0 (6)	C26—C27—H20	120.494
N1—C7—C6	121.0 (4)	C28—C27—H20	120.495
N1—C7—C8	115.3 (5)	C29—C30—H21	119.928
C6—C7—C8	123.7 (6)	C31—C30—H21	119.933
N2—C8—C7	115.3 (6)	C30—C31—H22	120.537
N2—C8—C9	121.3 (5)	C32—C31—H22	120.538
C7—C8—C9	123.3 (6)	C31—C32—H23	120.452
C8—C9—C10	119.8 (7)	C33—C32—H23	120.448
C9—C10—C11	119.1 (7)	N6—C33—H24	118.798
C10—C11—C12	119.1 (6)	C32—C33—H24	118.810
N2—C12—C11	122.4 (7)	N7—C34—H25	118.728
N3—C13—C14	123.0 (6)	C35—C34—H25	118.731
C13—C14—C15	118.9 (5)	C34—C35—H26	120.408
C14—C15—C16	118.6 (6)	C36—C35—H26	120.430
C15—C16—C17	120.3 (6)	C35—C36—H27	120.379
N3—C17—C16	120.7 (4)	C37—C36—H27	120.380
N3—C17—C18	115.2 (5)	C36—C37—H28	120.493
C16—C17—C18	124.0 (6)	C38—C37—H28	120.485
N4—C18—C17	115.7 (6)	C39—C40—H29	120.220
N4—C18—C19	121.8 (5)	C41—C40—H29	120.209
C17—C18—C19	122.4 (6)	C40—C41—H30	120.321
C18—C19—C20	118.8 (8)	C42—C41—H30	120.327
C19—C20—C21	119.2 (7)	C41—C42—H31	120.607
C20—C21—C22	119.6 (6)	C43—C42—H31	120.599
N4—C22—C21	121.8 (7)	N8—C43—H32	118.646
Ru2—C23—O4	177.9 (5)	C42—C43—H32	118.631
N5—C24—C25	122.7 (6)	C45—C44—H33	109.471
C24—C25—C26	118.8 (5)	C45—C44—H34	109.465
C25—C26—C27	119.4 (5)	C45—C44—H35	109.473
C26—C27—C28	119.0 (5)	H33—C44—H34	109.479
N5—C28—C27	121.3 (4)	H33—C44—H35	109.475
N5—C28—C29	115.2 (5)	H34—C44—H35	109.465
C27—C28—C29	123.6 (5)	O5—C46—H36	104.825
N6—C29—C28	115.3 (5)	O5—C46—H37	104.829
N6—C29—C30	120.3 (4)	O5^i^—C46—H36	127.377
C28—C29—C30	124.4 (5)	O5^i^—C46—H37	113.162
C29—C30—C31	120.1 (6)	C47—C46—H36	104.824
C30—C31—C32	118.9 (6)	C47—C46—H37	104.828
C31—C32—C33	119.1 (5)	H36—C46—H37	105.787
N6—C33—C32	122.4 (6)	C46—C47—H38	109.469
N7—C34—C35	122.5 (5)	C46—C47—H39	109.461
C34—C35—C36	119.2 (7)	C46—C47—H40	109.471
C35—C36—C37	119.2 (6)	H38—C47—H39	109.482
C36—C37—C38	119.0 (5)	H38—C47—H40	109.472
N7—C38—C37	122.0 (6)	H39—C47—H40	109.472
N7—C38—C39	115.5 (5)		
			
N1—Ru1—N2—C8	2.2 (4)	Ru1—N2—C12—C11	−176.8 (4)
N1—Ru1—N2—C12	180.0 (5)	C8—N2—C12—C11	0.9 (10)
N2—Ru1—N1—C3	174.8 (4)	C12—N2—C8—C7	177.2 (6)
N2—Ru1—N1—C7	1.1 (3)	C12—N2—C8—C9	0.5 (10)
N1—Ru1—N3—C13	93.4 (3)	Ru1—N3—C13—C14	−178.0 (3)
N1—Ru1—N3—C17	−85.6 (3)	Ru1—N3—C17—C16	177.3 (3)
N3—Ru1—N1—C3	−13.4 (4)	Ru1—N3—C17—C18	−3.4 (5)
N3—Ru1—N1—C7	172.9 (3)	C13—N3—C17—C16	−1.8 (6)
N1—Ru1—N4—C18	96.0 (3)	C13—N3—C17—C18	177.6 (4)
N1—Ru1—N4—C22	−87.8 (4)	C17—N3—C13—C14	1.0 (7)
N4—Ru1—N1—C3	−91.6 (4)	Ru1—N4—C18—C17	−0.6 (5)
N4—Ru1—N1—C7	94.7 (3)	Ru1—N4—C18—C19	177.7 (3)
C2—Ru1—N1—C3	83.0 (4)	Ru1—N4—C22—C21	−176.0 (3)
C2—Ru1—N1—C7	−90.7 (3)	C18—N4—C22—C21	0.0 (7)
N2—Ru1—N4—C18	172.6 (3)	C22—N4—C18—C17	−177.1 (4)
N2—Ru1—N4—C22	−11.2 (4)	C22—N4—C18—C19	1.2 (7)
N4—Ru1—N2—C8	−86.3 (4)	Ru2—N5—C24—C25	−179.6 (3)
N4—Ru1—N2—C12	91.5 (4)	Ru2—N5—C28—C27	179.3 (3)
N2—Ru1—C1—O1	121.0 (3)	Ru2—N5—C28—C29	−0.1 (5)
N2—Ru1—C1—O2	−57.2 (4)	C24—N5—C28—C27	−1.8 (6)
C1—Ru1—N2—C8	−179.0 (4)	C24—N5—C28—C29	178.7 (4)
C1—Ru1—N2—C12	−1.2 (5)	C28—N5—C24—C25	1.7 (7)
C2—Ru1—N2—C8	96.5 (4)	Ru2—N6—C29—C28	−2.9 (5)
C2—Ru1—N2—C12	−85.7 (4)	Ru2—N6—C29—C30	176.2 (3)
N3—Ru1—N4—C18	−0.9 (3)	Ru2—N6—C33—C32	−176.7 (3)
N3—Ru1—N4—C22	175.3 (4)	C29—N6—C33—C32	−0.9 (7)
N4—Ru1—N3—C13	−178.6 (3)	C33—N6—C29—C28	−179.0 (4)
N4—Ru1—N3—C17	2.4 (3)	C33—N6—C29—C30	0.1 (6)
N3—Ru1—C1—O1	−51.0 (3)	Ru2—N7—C34—C35	171.1 (3)
N3—Ru1—C1—O2	130.8 (4)	Ru2—N7—C38—C37	−170.5 (3)
C1—Ru1—N3—C13	−86.4 (3)	Ru2—N7—C38—C39	8.3 (5)
C1—Ru1—N3—C17	94.6 (3)	C34—N7—C38—C37	2.9 (6)
C2—Ru1—N3—C13	−2.2 (3)	C34—N7—C38—C39	−178.2 (4)
C2—Ru1—N3—C17	178.9 (3)	C38—N7—C34—C35	−1.7 (7)
N4—Ru1—C1—O1	27.4 (3)	Ru2—N8—C39—C38	2.8 (5)
N4—Ru1—C1—O2	−150.9 (4)	Ru2—N8—C39—C40	−176.1 (3)
C1—Ru1—N4—C18	−91.0 (3)	Ru2—N8—C43—C42	176.2 (3)
C1—Ru1—N4—C22	85.2 (4)	C39—N8—C43—C42	−2.5 (7)
C2—Ru1—C1—O1	−146.7 (4)	C43—N8—C39—C38	−178.3 (4)
C2—Ru1—C1—O2	35.1 (4)	C43—N8—C39—C40	2.8 (6)
O1—Ru2—N6—C29	−166.8 (3)	N1—C3—C4—C5	1.1 (8)
O1—Ru2—N6—C33	9.0 (3)	C3—C4—C5—C6	−1.7 (8)
N6—Ru2—O1—C1	−134.0 (3)	C4—C5—C6—C7	1.6 (9)
O1—Ru2—N7—C34	−96.4 (3)	C5—C6—C7—N1	−0.9 (8)
O1—Ru2—N7—C38	76.5 (3)	C5—C6—C7—C8	177.3 (5)
N7—Ru2—O1—C1	54.6 (3)	N1—C7—C8—N2	5.8 (8)
O1—Ru2—N8—C39	−90.1 (3)	N1—C7—C8—C9	−177.6 (5)
O1—Ru2—N8—C43	91.2 (3)	C6—C7—C8—N2	−172.4 (5)
N8—Ru2—O1—C1	132.7 (3)	C6—C7—C8—C9	4.2 (10)
C23—Ru2—O1—C1	−41.4 (3)	N2—C8—C9—C10	−1.8 (11)
N5—Ru2—N6—C29	2.2 (3)	C7—C8—C9—C10	−178.2 (6)
N5—Ru2—N6—C33	178.0 (4)	C8—C9—C10—C11	1.6 (11)
N6—Ru2—N5—C24	−179.8 (4)	C9—C10—C11—C12	−0.2 (11)
N6—Ru2—N5—C28	−1.0 (3)	C10—C11—C12—N2	−1.1 (11)
N5—Ru2—N7—C34	96.1 (3)	N3—C13—C14—C15	0.4 (7)
N5—Ru2—N7—C38	−91.0 (3)	C13—C14—C15—C16	−1.0 (8)
N7—Ru2—N5—C24	−8.4 (4)	C14—C15—C16—C17	0.3 (8)
N7—Ru2—N5—C28	170.4 (3)	C15—C16—C17—N3	1.1 (8)
N5—Ru2—N8—C39	101.5 (3)	C15—C16—C17—C18	−178.1 (5)
N5—Ru2—N8—C43	−77.2 (3)	N3—C17—C18—N4	2.6 (6)
N8—Ru2—N5—C24	−86.0 (3)	N3—C17—C18—C19	−175.7 (4)
N8—Ru2—N5—C28	92.8 (3)	C16—C17—C18—N4	−178.1 (5)
C23—Ru2—N5—C24	88.4 (4)	C16—C17—C18—C19	3.6 (8)
C23—Ru2—N5—C28	−92.8 (3)	N4—C18—C19—C20	−1.7 (8)
N6—Ru2—N8—C39	179.7 (3)	C17—C18—C19—C20	176.5 (4)
N6—Ru2—N8—C43	1.0 (3)	C18—C19—C20—C21	0.9 (8)
N8—Ru2—N6—C29	−85.0 (3)	C19—C20—C21—C22	0.2 (9)
N8—Ru2—N6—C33	90.9 (3)	C20—C21—C22—N4	−0.7 (9)
C23—Ru2—N6—C29	95.8 (3)	N5—C24—C25—C26	−0.1 (8)
C23—Ru2—N6—C33	−88.4 (4)	C24—C25—C26—C27	−1.4 (8)
N7—Ru2—N8—C39	1.2 (3)	C25—C26—C27—C28	1.2 (7)
N7—Ru2—N8—C43	−177.6 (3)	C26—C27—C28—N5	0.4 (7)
N8—Ru2—N7—C34	−178.2 (3)	C26—C27—C28—C29	179.8 (4)
N8—Ru2—N7—C38	−5.3 (3)	N5—C28—C29—N6	2.0 (6)
C23—Ru2—N7—C34	1.0 (4)	N5—C28—C29—C30	−177.0 (4)
C23—Ru2—N7—C38	173.9 (3)	C27—C28—C29—N6	−177.4 (4)
Ru2—O1—C1—Ru1	−173.50 (17)	C27—C28—C29—C30	3.5 (7)
Ru2—O1—C1—O2	4.8 (6)	N6—C29—C30—C31	0.5 (7)
O5^i^—O5—C46—O5^i^	0 (2)	C28—C29—C30—C31	179.5 (4)
O5^i^—O5—C46—C47	14 (5)	C29—C30—C31—C32	−0.3 (7)
C46—O5—O5^i^—C46	0.0 (11)	C30—C31—C32—C33	−0.6 (8)
O5^i^—O5—C46^i^—O5^i^	−0 (3)	C31—C32—C33—N6	1.2 (8)
O5^i^—O5—C46^i^—C47^i^	169 (4)	N7—C34—C35—C36	−0.4 (8)
C46^i^—O5—O5^i^—C46^i^	0.0 (11)	C34—C35—C36—C37	1.1 (8)
C46—O5—C46^i^—O5^i^	−0 (4)	C35—C36—C37—C38	0.1 (8)
C46—O5—C46^i^—C47^i^	169 (5)	C36—C37—C38—N7	−2.2 (7)
C46^i^—O5—C46—O5^i^	0 (3)	C36—C37—C38—C39	179.0 (4)
C46^i^—O5—C46—C47	14 (7)	N7—C38—C39—N8	−7.3 (6)
Ru1—N1—C3—C4	−173.8 (3)	N7—C38—C39—C40	171.5 (4)
Ru1—N1—C7—C6	174.4 (3)	C37—C38—C39—N8	171.6 (4)
Ru1—N1—C7—C8	−3.9 (5)	C37—C38—C39—C40	−9.6 (7)
C3—N1—C7—C6	0.2 (7)	N8—C39—C40—C41	−1.2 (7)
C3—N1—C7—C8	−178.1 (4)	C38—C39—C40—C41	−179.9 (4)
C7—N1—C3—C4	−0.3 (8)	C39—C40—C41—C42	−0.9 (8)
Ru1—N2—C8—C7	−4.9 (8)	C40—C41—C42—C43	1.2 (8)
Ru1—N2—C8—C9	178.4 (5)	C41—C42—C43—N8	0.5 (8)

Symmetry codes: (i) −*x*−1, −*y*+1, −*z*; (ii) −*x*+1, −*y*, −*z*+1; (iii) −*x*+1, −*y*+1, −*z*+1; (iv) −*x*, −*y*+1, −*z*+1; (v) −*x*+1, −*y*, −*z*+2; (vi) *x*+1, *y*, *z*; (vii) −*x*, −*y*, −*z*+2; (viii) *x*, *y*, *z*+1; (ix) *x*−1, *y*+1, *z*−1; (x) −*x*, −*y*, −*z*+1; (xi) −*x*, −*y*+1, −*z*; (xii) *x*+1, *y*−1, *z*; (xiii) *x*+1, *y*−1, *z*+1; (xiv) *x*, *y*−1, *z*; (xv) *x*−1, *y*, *z*; (xvi) *x*, *y*, *z*−1; (xvii) *x*, *y*+1, *z*; (xviii) *x*−1, *y*+1, *z*; (xix) *x*, *y*+1, *z*−1; (xx) −*x*−1, −*y*+1, −*z*+1; (xxi) *x*, *y*−1, *z*+1.

### Hydrogen-bond geometry (Å, º)

**Table:**

*D*—H···*A*	*D*—H	H···*A*	*D*···*A*	*D*—H···*A*
C5—H3···F5*A*^xii^	0.95	2.54	3.390 (8)	149
C5—H3···F5*B*^xii^	0.95	2.18	2.91 (4)	133
C6—H4···F4*B*^iii^	0.95	2.52	2.88 (3)	102
C11—H7···F6*A*	0.95	2.44	3.269 (8)	145
C12—H8···O2	0.95	2.49	3.241 (9)	136
C13—H9···O2^v^	0.95	2.39	3.105 (6)	132
C19—H13···F4*A*^xiv^	0.95	2.29	3.077 (9)	140
C21—H15···F7	0.95	2.30	3.237 (10)	170
C25—H18···F12^iv^	0.95	2.44	3.130 (8)	129
C30—H21···O4^vii^	0.95	2.56	3.473 (7)	162
C33—H24···O1	0.95	2.47	3.035 (7)	118
C36—H27···F1	0.95	2.40	3.292 (8)	156
C36—H27···F3*A*	0.95	2.53	3.322 (9)	141
C36—H27···F3*B*	0.95	2.48	3.16 (4)	128
C37—H28···F11	0.95	2.49	3.205 (9)	132
C42—H31···F5*A*^xiv^	0.95	2.50	3.320 (8)	145
C43—H32···F2^xiv^	0.95	2.48	3.223 (7)	135
C44—H34···F7	0.98	2.54	3.318 (10)	136
C47—H39···F6*A*^iv^	0.98	2.30	3.18 (3)	148
C47—H39···F3*B*^iv^	0.98	2.39	3.19 (4)	139

Symmetry codes: (iii) −*x*+1, −*y*+1, −*z*+1; (iv) −*x*, −*y*+1, −*z*+1; (v) −*x*+1, −*y*, −*z*+2; (vii) −*x*, −*y*, −*z*+2; (xii) *x*+1, *y*−1, *z*; (xiv) *x*, *y*−1, *z*.
